# Mechanosensory and ATP Release Deficits following Keratin14-Cre-Mediated TRPA1 Deletion Despite Absence of TRPA1 in Murine Keratinocytes

**DOI:** 10.1371/journal.pone.0151602

**Published:** 2016-03-15

**Authors:** Katherine J. Zappia, Sheldon R. Garrison, Oleg Palygin, Andy D. Weyer, Marie E. Barabas, Michael W. Lawlor, Alexander Staruschenko, Cheryl L. Stucky

**Affiliations:** 1 Department of Cell Biology, Neurobiology and Anatomy, Medical College of Wisconsin, Milwaukee, Wisconsin, United States of America; 2 Department of Physiology, Medical College of Wisconsin, Milwaukee, Wisconsin, United States of America; 3 Division of Pediatric Pathology, Department of Pathology, Medical College of Wisconsin, Milwaukee, Wisconsin, United States of America; Toronto University, CANADA

## Abstract

Keratinocytes are the first cells that come into direct contact with external tactile stimuli; however, their role in touch transduction *in vivo* is not clear. The ion channel Transient Receptor Potential Ankyrin 1 (TRPA1) is essential for some mechanically-gated currents in sensory neurons, amplifies mechanical responses after inflammation, and has been reported to be expressed in human and mouse skin. Other reports have not detected *Trpa1* mRNA transcripts in human or mouse epidermis. Therefore, we set out to determine whether selective deletion of *Trpa1* from keratinocytes would impact mechanosensation. We generated *K14Cre-Trpa1*^fl/fl^ mice lacking TRPA1 in K14-expressing cells, including keratinocytes. Surprisingly, *Trpa1* transcripts were very poorly detected in epidermis of these mice or in controls, and detection was minimal enough to preclude observation of *Trpa1* mRNA knockdown in the *K14Cre-Trpa1*^fl/fl^ mice. Unexpectedly, these *K14Cre-Trpa1*^fl/fl^ mice nonetheless exhibited a pronounced deficit in mechanosensitivity at the behavioral and primary afferent levels, and decreased mechanically-evoked ATP release from skin. Overall, while these data suggest that the intended targeted deletion of *Trpa1* from keratin 14-expressing cells of the epidermis induces functional deficits in mechanotransduction and ATP release, these deficits are in fact likely due to factors *other* than reduction of *Trpa1* expression in adult mouse keratinocytes because they express very little, if any, *Trpa1*.

## Introduction

Keratinocytes of the epidermis have traditionally been viewed as passive, protective barrier cells that shield the body against environmental stressors. However, keratinocytes are also the first contact point for external mechanical stimuli, and only recently has their potential contribution to touch transduction received attention [1–4]. As such, keratinocytes are emerging as pivotal members of the somatosensory response because they are highly innervated by free nerve endings and are adjacent to mechanosensitive Merkel cells, Meissner’s corpuscles, and hair follicles. Due to their proximity to both the environment and to other mechanosensory structures, keratinocytes are well situated to communicate with neurons and other non-neural cells that contribute to dynamic tactile and thermal responses. In particular, keratinocytes are closely apposed to sensory nerve terminals, especially those belonging to C fiber afferents corresponding to isolectin B4-binding (IB4^+^) small-diameter neurons [5]. Keratinocytes in isolation have been shown to respond directly to force *in vitro* [1,2,6,7]; however, experimental data *in vivo* are lacking and the mechanisms are poorly understood.

Cultured keratinocytes respond directly to multiple somatosensory stimuli, including force [2,8]. Keratinocytes also respond directly to heat (>40°C) via transient receptor potential vanilloid 3 (TRPV3). Furthermore, TRPV3 activation in keratinocytes mediates release of ATP, which then activates ATP-sensitive P2Y receptors on dorsal root ganglion (DRG) nerve terminals [9]. Moreover, the response in keratinocytes can be amplified via gap-junctional intercellular Ca^2+^ and ATP waves that propagate through keratinocytes [2,6,10], broadening the capability for release of signaling molecules. However, the mechanisms of keratinocytes responding to mechanical or cold sensation remain unknown, and have not been explored *in vivo*. Therefore, we sought to provide insight into how these cells communicate with other sensory structures in the skin to induce tactile and thermal responses.

We began by focusing on the non-selective cation channel TRPA1 (transient receptor potential ankyrin 1) because it plays an important role in touch and pain responses [11–13]. While TRPA1 is strongly expressed in adult sensory neurons [13,14], there are reports that TRPA1 is also expressed in keratinocytes [8,13,15]. Some of this expression data was obtained from neonatal human tissue, which does not necessarily imply that *Trpa1* is expressed in mouse keratinocytes. Moreover, additional recent evidence suggests that *Trpa1* is not expressed in murine keratinocytes isolated from neck skin [16]. Therefore we sought to determine both the expression patterns and functionality of *Trpa1* in mouse keratinocytes from hindpaw skin. To separate the contribution of keratinocytes from neurons, we utilized *K14Cre* mice to express Cre recombinase in keratin 14-expressing keratinocytes [17]. We also generated a novel mouse in which loxP sites flank the exons encoding the pore region of TRPA1 (*Trpa1*^fl/fl^). Crossing these two mouse lines generated *K14Cre-Trpa1*^fl/fl^ mice. Although the keratinocytes isolated from even control mice did not contain detectable *Trpa1* transcripts, the mutant mice exhibited sensory deficits at behavioral and afferent levels, as well as decreases in mechanically-evoked ATP release from the skin.

## Results

### Expression of Trpa1 Is Not Detected in Mouse Epidermis

**Fig 1:**
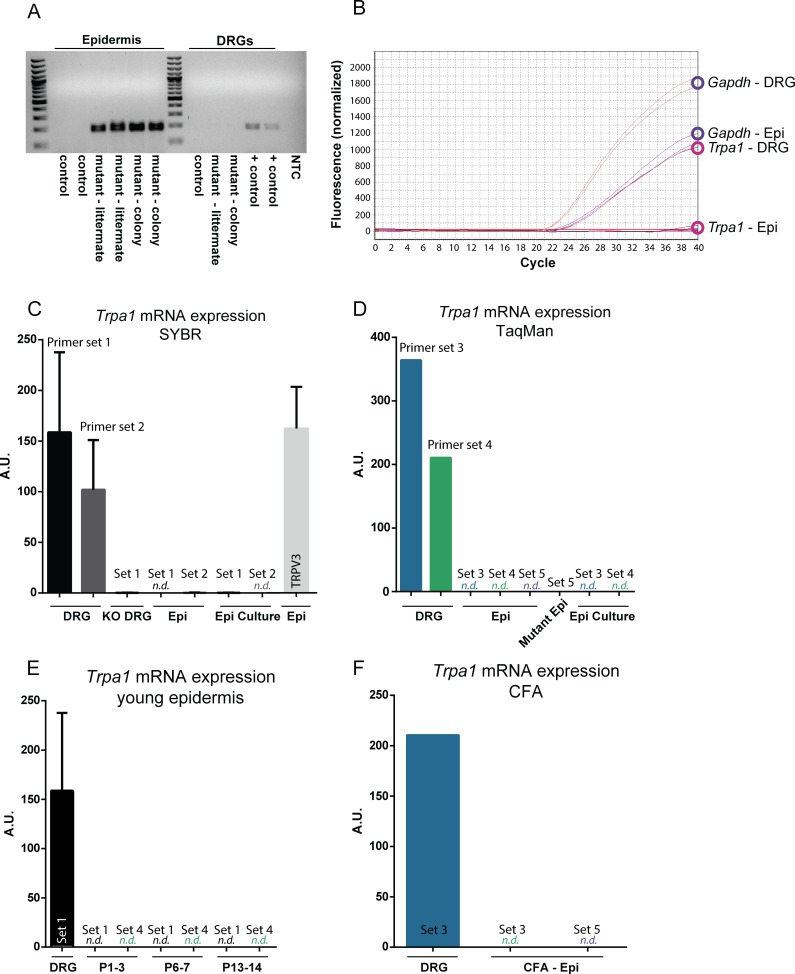
*Trpa1* mRNA is poorly detected in mouse epidermal keratinocytes. (**A**) Following Cre-mediated recombination of genomic *Trpa1* DNA, an excision product is amplified in the epidermis of *K14Cre*-*Trpa1*^fl/fl^ mice (left), but not in the control (*K14Cre-Trpa1*^*+/+*^) mice. Mutant mice (*K14Cre-Trpa1*^*fl/fl*^) mice were either from an independently maintained colony (mutant-colony), or generated as littermates from heterozygous breeders (mutant—littermate). No excision product was observed in the DRGs of either control or mutant mice. Positive control band was obtained from DRG tissue from an *AdvillinCre-Trpa1*^*fl/fl*^ mouse (right). For panels B-F, mRNA was isolated from DRG and epidermis from control mice. (**B**) Amplification plot showing *Gapdh* and *Trpa1* mRNA transcript amplification. *Gapdh* was consistently detected and quantified in both DRG and epidermal samples (top). *Trpa1* mRNA was strongly detected in DRG samples; however, the same PCR protocol did not detect measurable *Trpa1* in epidermal samples (bottom). (**C**) Two different primer sets to detect *Trpa1* were effective in measuring *Trpa1* from DRG samples using SYBR Green qPCR, but did not amplify *Trpa1* from epidermal samples or cultured epidermal keratinocytes. Primer set 1 targets exons 22–23 within the deleted pore region of *Trpa1*; primer set 2 targets exons 17–19, upstream of the deleted pore region. (**D**) Three sets of Taqman primer-probes were similarly unable to detect *Trpa1* transcripts in control epidermis. Primer set 3 targets exons 13–14, set 4 targets exons 22–23, and set 5 targets exons 23–24 of *Trpa1*. (**E**) Neither qPCR for exons 22–23 (set 1) nor exons 22–23 (set 4) were capable of detecting *Trpa1* in neonatal mouse epidermis. (**F**) Two days after hindpaw injection of CFA, *Trpa1* remained undetected in epidermis. *N*.*d*. denotes transcript not detected.

Due to discrepancies in the literature regarding whether *Trpa1* is expressed in keratinocytes of the epidermis [8,13,15,16], we became interested in confirming its expression in mouse epidermis. With a goal of isolating the effects of TRPA1 in keratinocytes, we used a *K14Cre mouse* expressing Cre recombinase under the keratin 14 promoter [18]. Since keratin 14 is expressed in cells of the ectoderm [17], Cre recombinase will thus be expressed in all keratinocytes of the epidermis, as well as in sebaceous and sweat glands, but will be absent from mesodermal-derived cells such as fibroblasts or other cells of the dermis and subcutaneous tissues. We mated *K14Cre* mice with *Trpa1*^fl/fl^ mice to generate *K14Cre-Trpa1*^fl/fl^ (mutant) mice (**S1 Fig**). *K14Cre-Trpa1*^*+/+*^ mice were used as controls. It was confirmed that Cre-mediated recombination created an excision product specifically within the epidermis of Cre^+^ animals (**Fig 1A**). In addition to confirming the expression patterns of *Trpa1* in control mouse epidermis, it was also necessary to evaluate the efficiency and selectivity of the knockout of *Trpa1* within keratin 14-expressing cells.

Since the epidermis is comprised of roughly 95% keratinocytes [19], and since keratin 14 has largely been accepted as a keratinocyte-selective marker, we isolated mRNA from the glabrous hindpaw epidermis of control mice. To our surprise, *Trpa1* mRNA was undetectable in control mouse epidermis (**Fig 1B and 1C**). We questioned whether the primer set initially used was not ideally suited for low copy number transcripts or whether it did not target a stable part of the *Trpa1* mRNA sequence despite qPCR protocol optimization; therefore, we repeated this assay using an additional primer set designed to target exons upstream of the pore regions (primer set 2; **Fig 1C**). While no *Trpa1* was reliably detected in mRNA isolated from epidermal samples, *Trpa1* transcripts were easily detected in DRGs. Further, other genes including *Trpv3*, known to be expressed in keratinocytes [20], and also a common housekeeping gene, *Gapdh*, were both consistently detected in mRNA isolated from the epidermal samples (**Fig 1B and 1C**). We next attempted to measure *Trpa1* expression in skin using three TaqMan probe-based assays for gene expression analysis (LifeTechnologies), as these assays have been externally validated and the inclusion of a sequence-specific probe could enhance our ability to detect *Trpa1*-specific signal, even at low expression levels (primer sets 3, 4, 5; defined in **S1 Table**, **Fig 1D**). Again, these assays showed minimal to no expression of *Trpa1* in the epidermis. Overall, these data are consistent with the recent Liu *et al*. report that wildtype mouse epidermis does not contain detectable *Trpa1* transcripts [16].

We next considered that, despite an apparent lack of *Trpa1* expression in adult skin, there might have been *Trpa1* expression in keratinocytes early in development to initiate or ultimately modulate keratinocyte function in mechanosensation. We obtained skin samples from a range of embryonic stages, including E10.5, E12.5, E14.5 and E16.5, from the *K14Cre-tdTomato* reporter mice. tdTomato-expressing keratinocytes were dissociated and isolated via fluorescence-activated cell sorting (FACS), and qPCR was performed on RNA isolated from these cells. No *Trpa1* expression was reliably observed in these samples from control or mutant animals at any developmental stage (not shown). Further RT-PCR of samples isolated from glabrous epidermis at ages P1 through P14 similarly revealed a lack of *Trpa1* mRNA, consistent with our findings in adult epidermal tissues (**Fig 1E**). We also considered the possibility that *Trpa1* mRNA levels were maintained tonically in the epidermis at such low levels that they are essentially undetectable. Others have demonstrated elevations in *Trpa1* mRNA expression in the DRG following an inflammatory injury [21], so we next asked whether an acute inflammation could raise levels of *Trpa1* mRNA in the epidermis into the detectable range. However, even 2 days after CFA inflammation in the hindpaw, no *Trpa1* mRNA was detected in the epidermis (**Fig 1F**).

**Fig 2:**
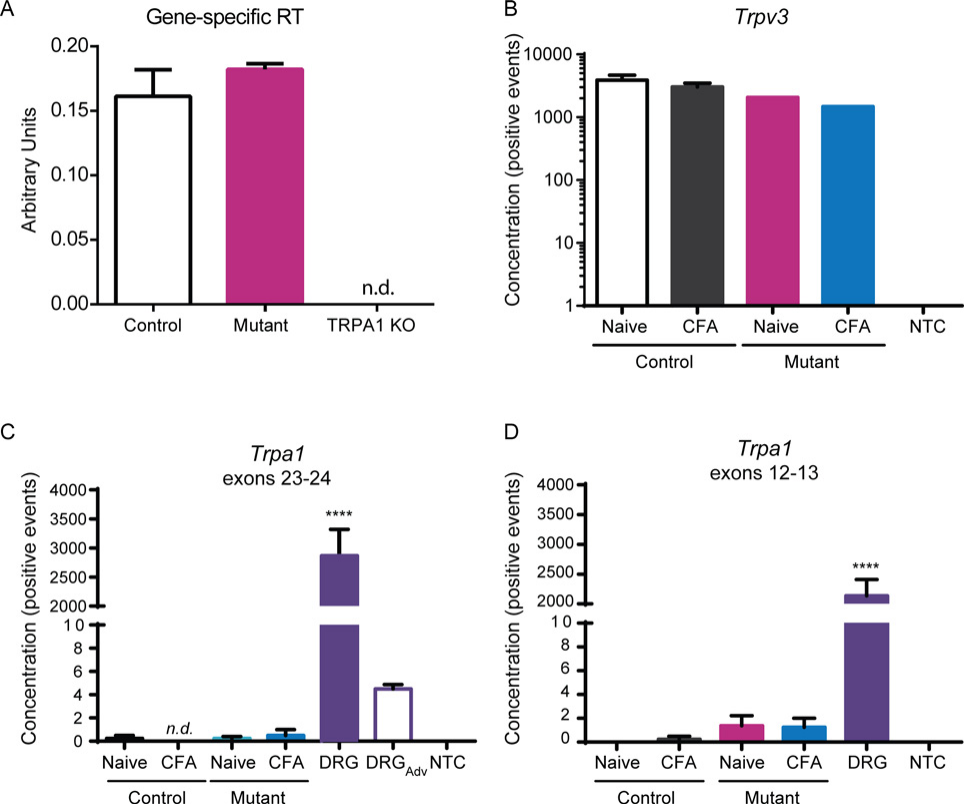
No *Trpa1* knockdown is observed in epidermis of mutant (*K14Cre-Trpa1*^*fl/fl*^) mice compared to controls (*K14Cre-Trpa1*^*+/+*^). (**A**) Gene-specific primers to improve reverse transcription of *Trpa1* were used prior to targeted amplification of *Trpa1*. This sensitive method detected a very small amount of *Trpa1* transcripts within hindpaw epidermis, and the amplification was equivalent between control and mutant samples. (**B-D**) Quantitative digital-droplet PCR was performed on samples isolated from epidermis of control and mutant mice. (**B**) TRPV3 was efficiently detected in all samples tested. No template control (NTC) bars denote ddPCR performed with no cDNA added. (**C**) ddPCR for exons 23–24 of *Trpa1* (within the loxp-flanked region) detected ample transcripts within the DRG samples. On average, less than one positive event per entire reaction volume was detected in both control and mutant samples. A control sample of DRG tissue from an *AdvillinCre-Trpa1*^fl/fl^ mouse shows that Cre-mediated recombination in a different tissue (sensory neurons) reduces *Trpa1* mRNA detection (DRG_Adv_). (**D**) ddPCR for exons 12–13 detected on average less than one positive event per epidermal sample. *N*.*d*. denotes transcript not detected. **** *P<*0.0001, compared to every other bar.

The only method that could more consistently detect *Trpa1* transcripts involved gene-specific primers for reverse transcription prior to qPCR for *Trpa1* to increase our possible chances of amplifying any *Trpa1* transcripts (**Fig 2A**). Here, *Trpa1* was observed at low levels in both control *K14Cre-Trpa1*^fl/fl^ and mutant *K14Cre-Trpa1*^fl/fl^ epidermis, but was undetected in a global *Trpa1* knockout mouse. Even then, due the incredibly low levels of *Trpa1*, we were unable to observe a difference in *Trpa1* transcripts between the control and *K14Cre-Trpa1*^fl/fl^ epidermis. Further, additional experiments using *in situ* hybridization were unable to detect *Trpa1* in the epidermis, and likewise could not distinguish between control and mutant tissues (data not shown). Therefore, either the current range of detection may be too low to observe or quantitate any knockdown of *Trpa1*, or perhaps more likely, the few transcripts detected may have arisen from other cell types within the epidermal preparations.

In a continued search for *Trpa1* expression in mouse keratinocytes, we next used digital droplet PCR (ddPCR), an additional sensitive assay that can be used to detect very low mRNA expression levels. Efficacy of the procedure was first addressed by obtaining reasonable expression levels of *Trpv3* in all samples tested (**Fig 2B**). As in traditional qPCR, this assay revealed minimal to no expression of *Trpa1* in the epidermis, while it detected a very high expression of *Trpa1* in DRGs, as expected (**Fig 2C and 2D**). Epidermal samples had on average less than one single positive event representing *Trpa1* expression per sample. Importantly, this single event was the derived from the cumulative mRNA isolated from *thousands* of keratinocytes.

### K14Cre-Trpa1^fl/fl^ Mutant Mice Exhibit Impaired Mechanosensitivity

**Fig 3:**
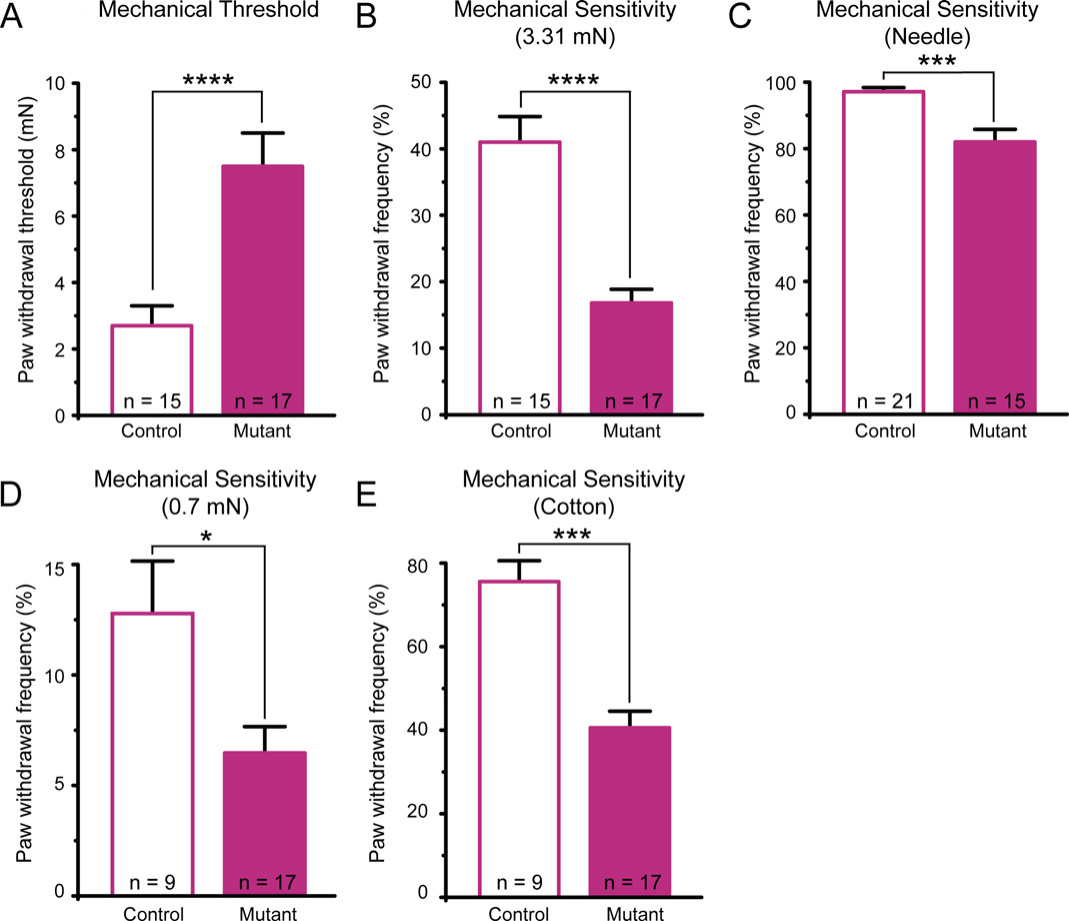
Mutant mice (*K14Cre-Trpa1*^fl/fl^) exhibit decreased behavioral sensitivity to noxious and gentle mechanical stimuli. (**A**) Paw withdrawal responses revealed markedly elevated thresholds in mutant mice. (**B**) Mutant mice had significantly fewer responses to repeated application of a 3.31 mN von Frey filament. (**C**) Mutant mice responded less frequently to repeated applications of a spinal needle. (**D**). Mutant mice responded less frequently to repeated, punctate application of a 0.7 mN von Frey filament to the plantar hindpaw. (**E**) Mutant mice exhibited decreased responses to gentle stroking puffed-out cotton swab applied to the hindpaw. * *P*<0.05; *** *P*<0.001, **** *P<*0.0001. Data reported as mean ± s.e.m.

Despite observing an often-undetected level of baseline *Trpa1* expression within control mouse epidermis, and consequently no further reduction of *Trpa1* mRNA expression in mutant epidermis, we surprisingly still observed a prominent phenotypic deficit in the mutant mice. First, we observed that the *K14Cre-Trpa1*^fl/fl^ mutant mice have a striking deficit in behavioral mechanosensation. Mutant mice displayed decreased mechanical sensitivity by exhibiting a 2.8-fold increase in paw withdrawal thresholds (**Fig 3A**), a 60% decrease in response to a suprathreshold (3.31 mN) von Frey filament (**Fig 3B**), and a 15% decrease in response to a spinal needle prick (**Fig 3C**) compared to control mice. We also assessed responses to innocuous light touch. Testing behavioral responses to light touch applied to the plantar hindpaw, we found a 49% decrease in response to light punctate force (**Fig 3D**) and a 46% decrease in response to dynamic gentle stroke in mutant mice (**Fig 3E**).

**Fig 4:**
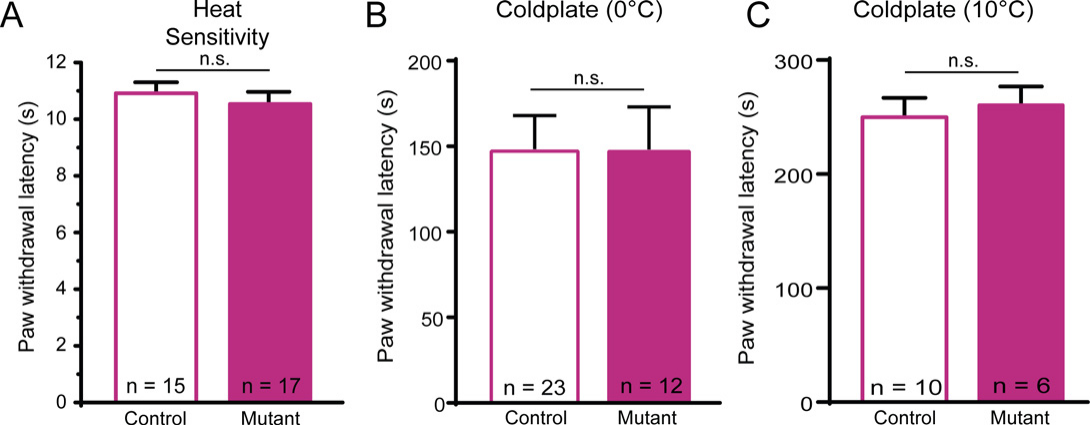
Mutant mice have normal behavioral thermal sensitivity. (**A**) Mutant and control mice did not differ in latency to respond to a heat lamp directed at the plantar hindpaw. (**B-C**) Mutant and control mice exhibited similar paw lift response latency to a cold plate at either 0°C (B) or 10°C (C). Data reported as mean ± s.e.m. *N*.*s*. denotes not significant.

In contrast, the mutant mice showed no deficits in thermal sensitivity. Behavioral responses to heat were not different between the mutants and controls (**Fig 4A**). Cold sensitivity was assessed by placing mice on either a 0° or 10°C cold surface for 5 minutes and quantifying response latency. There were no differences in response latency between mutant mice and controls at either 0°C and 10°C (**Fig 4B and 4C**). Together, these data suggest that the baseline sensory deficits in these mutants were restricted to mechanosensation.

**Fig 5:**
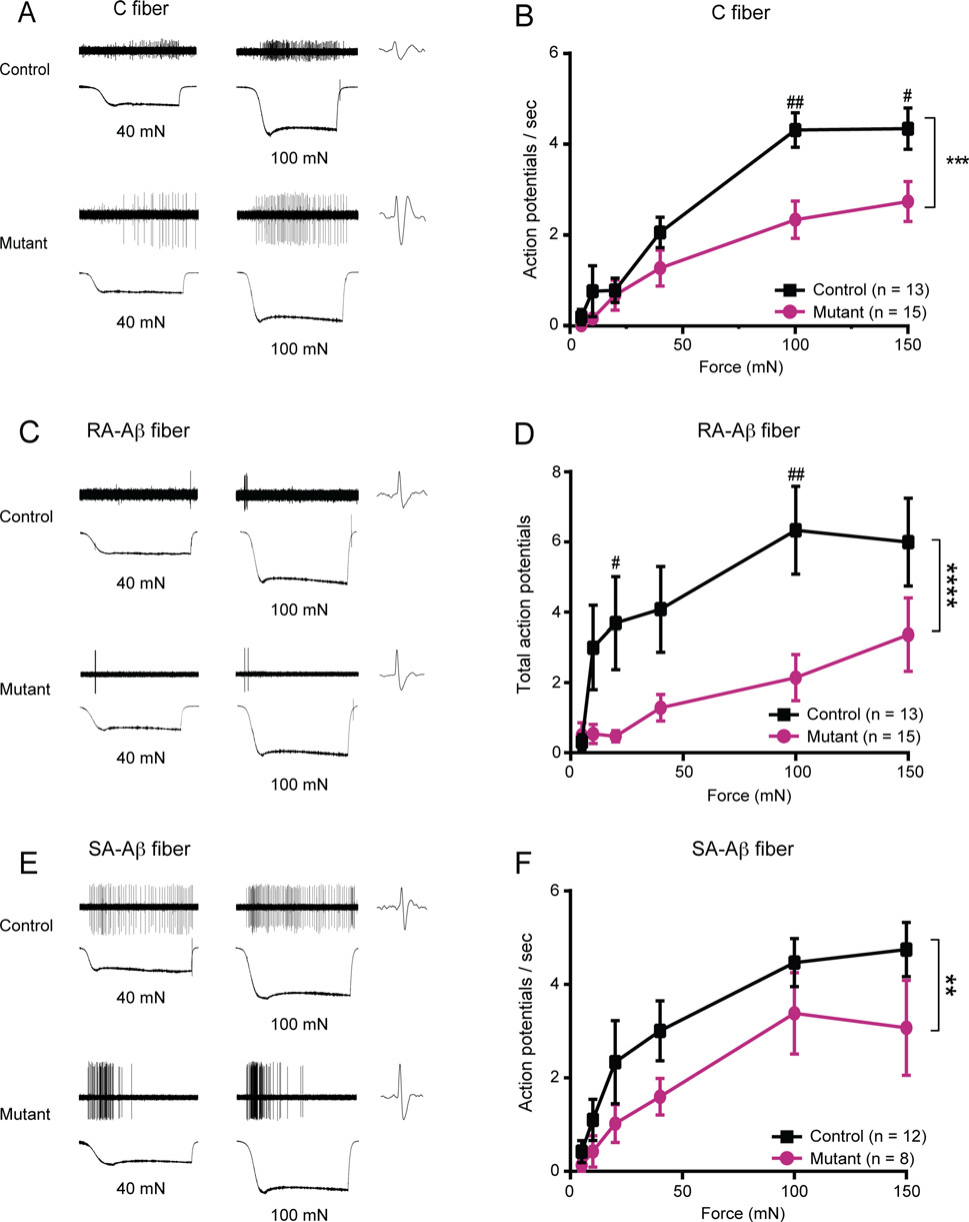
Mutant mice exhibited decreased mechanically-evoked action potential firing. Glabrous skin-sural nerve preparations were dissected from mutant and control mice. (**A**) Examples of responses of single C fiber nociceptors to 40 and 100 mN stimuli, shown for both a control fiber (top) and a mutant fiber (bottom). (**B**) Average action potential firing rate of mutant fibers compared to control was significantly reduced in C fibers innervating glabrous skin. (**C**) Sample RA-Aβ fiber responses from control (top) and mutant (bottom) glabrous skin recordings at forces of 40 and 100 mN. (**D**) Mutant RA-Aβ fibers fired significantly fewer total action potentials compared to control fibers. By 100 mN, there was a 73.8% reduction in the action potential firing rate in mutants compared to the firing rate in controls at 100 mN. (**E**) Example responses of a control (top) and mutant (bottom) SA-Aβ fiber in response to 40 and 100 mN stimulation. (**F**) Action potential firing rate in mutant SA-Aβ fibers was significantly decreased compared to control fibers, with a cumulative 31% reduction in action potentials fired across all forces tested. ** *P*<0.01; *** *P*<0.001, **** *P*<0.0001 comparing overall responses between control and mutant fibers. # *P*<0.05, ## *P*<0.01 comparing action potential firing at a single force. Data reported as mean ± s.e.m.

Since reductions in behavioral mechanosensitivity may be mediated by decreased activity of sensory afferents, we asked whether the firing of nociceptive peripheral sensory neurons was also impacted by the targeted deletion of *Trpa1* from K14-expressing cells. We used the *ex vivo* skin-nerve preparation, which allows the sensory terminals to remain in their native anatomical arrangement with keratinocytes and other skin cells *in situ*. We recorded action potentials in single sural nerve afferents that innervate the plantar glabrous skin tested in behavioral assays. C fiber nociceptors exhibited a pronounced decrease in action potential firing rates in mutant skin-nerve preparations at all forces tested, reaching a 46% reduction in action potentials fired at 100 mN (**Fig 5A and 5B**).

In agreement with the innocuous light touch behavior, *ex vivo* sural skin-nerve recordings revealed a marked deficit in light-touch afferent terminals. Rapidly-adapting Aβ (RA-Aβ) fibers, which innervate Meissner’s corpuscles in glabrous skin, from mutant mice fired fewer action potentials across all forces, for example showing a 66% percent reduction in total action potentials fired at 100 mN (**Fig 5C and 5D**). Furthermore, glabrous slowly-adapting Aβ (SA-Aβ) fibers, many of which innervate Merkel cells, exhibited a significant decrease in total mechanically-evoked action potentials as well (**Fig 5E**). No deficits in conduction velocity or von Frey thresholds were found for any fiber type in mutant mice (**S2 Table**).

### Inflammatory Hypersensitivity Is Impaired in Mutant Mice

**Fig 6:**
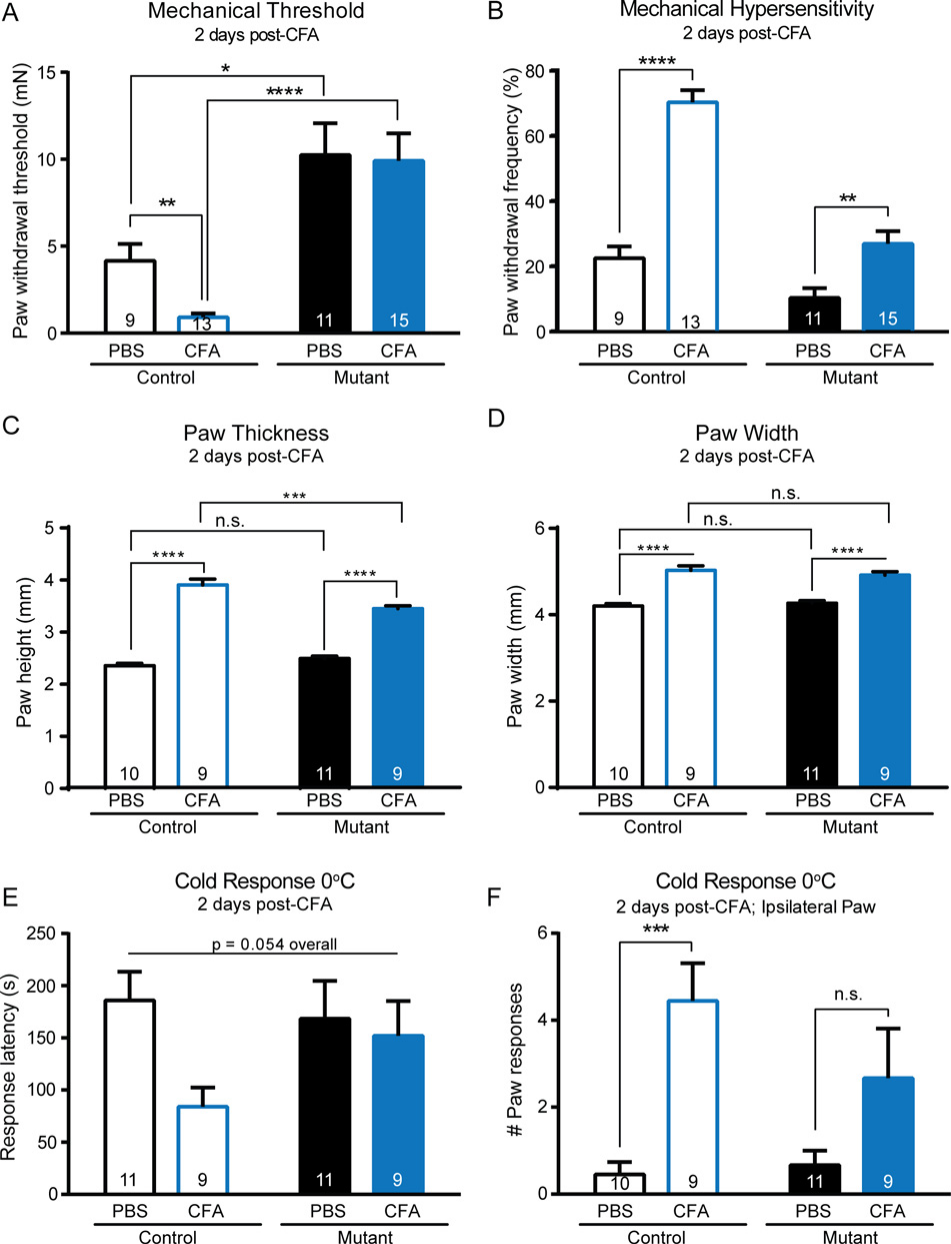
Mutant mice exhibit minimal inflammatory allodynia and hyperalgesia two days after CFA injection. (**A**) Following injection of CFA into the hindpaw, control mice exhibited a marked increase in mechanical sensitivity following CFA injection into the hindpaw as indicated by the reduction in paw withdrawal threshold. In contrast, no changes in threshold were observed in mutant mice treated with CFA. After inflammation, there remained a striking difference in paw withdrawal threshold between mutant and control animals. Analysis was performed using a 2-way ANOVA with *post hoc* analysis via Mann-Whitney *U* tests with Bonferroni adjustment. (**B**) As indicated by a significant increase in paw withdrawal frequency to repeated application of a 3.31 mN force (left), control mice displayed hypersensitivity after CFA injection. Mutant mice exhibited a smaller, yet significant, increase in withdrawal frequency (right). (**C**) CFA injection induced an increase in the injected paw thickness of both control and mutant mice. Following CFA, control paws were significantly thicker that mutant paws, though the effect size was limited. (**D**) Similarly, CFA increased paw width in both control and mutant mice. (**E**) There were no overall differences in paw withdrawal latency to a 0°C cold plate, though there was a strong trend suggesting CFA may have decreased cold withdrawal latency only in the control mice. (**F**) CFA greatly increased the *number* of paw lifts and responses to a 0°C cold plate in control mice, and did not significantly increase paw responses in mutant mice. * *P*<0.05, ** *P*<0.01; *** *P*<0.001, **** *P*<0.0001.

Since the results of the baseline characterization of the mechanosensitivity in this *K14Cre-Trpa1*^fl/fl^ mutant were striking, we next asked how inflammation might impact its behavioral and sensory physiology. In accordance with the decreased baseline sensitivity, we questioned whether the mutant mouse might also be protected from inflammatory hypersensitivity. Indeed, inflammation induced by hindpaw injection of CFA resulted in a marked reduction in inflammatory hypersensitivity in mutant mice when compared to control mice. In the CFA-injected control paw, we observed heightened mechanical sensitivity that included a 4.7-fold decrease in paw withdrawal threshold and a 3.1-fold increase in paw withdrawal frequency to repeated application of a suprathreshold force compared to saline-injected controls (**Fig 6A**). In contrast, CFA-injected mutant mice showed no change in paw withdrawal threshold compared to saline-injected mice (**Fig 6A**). However, these mice exhibited a small, significant increase in paw withdrawal frequency after inflammation, indicating a heightened responsiveness to repeated force (**Fig 6B**). CFA-injected mutant mice reached a withdrawal frequency of 26.7%, while control mice responded at a rate of 70%. Therefore even during inflammatory pain, mutant mice exhibited a 62% reduction in responses to mechanical stimuli compared to inflamed control mice, and showed only a mild mechanical sensitization following hindpaw inflammation. Importantly, CFA inflammation induced an increase in total paw thickness and width in both control and mutant mice (**Fig 6C and 6D**), which contrasts the limited impact of inflammation on mechanical threshold in the mutant mice. There were no differences in paw thickness or width in PBS injected animals. Following CFA-induced inflammation, mutant animals had a slight but significant reduction in paw thickness compared to controls, while width was similar between control and mutant mice after CFA (**Fig 6C and 6D**).

In addition, behavioral cold sensitivity was assessed in the mouse strains 2 days after CFA-induced inflammation (**Fig 6E and 6F**). In control mice injected with CFA, the response latency to 0°C cold was reduced 55% compared to saline-injected controls, though an overall two-way ANOVA did not detect statistical significance (*p* = 0.0538; **Fig 6E**). Additionally, CFA inflammation caused a sharp increase in the number of paw responses (lifts, flicks, raises) in the injected paw of control mice (**Fig 6F**). However, cold hypersensitivity appeared to be absent in CFA-injected mutant mice, as there was no significant difference in either initial response latency or number of paw responses in the saline-injected compared to CFA injected mutant mice (**Fig 6E and 6F**). Overall, the collective data thus far show that these mutant mice have a significant impairment to both noxious and innocuous mechanical stimuli in naïve conditions, and disrupted mechanical and cold sensitization after inflammation.

### Alternative Sources of Sensory Disruption in the Mutant Mice

**Fig 7:**
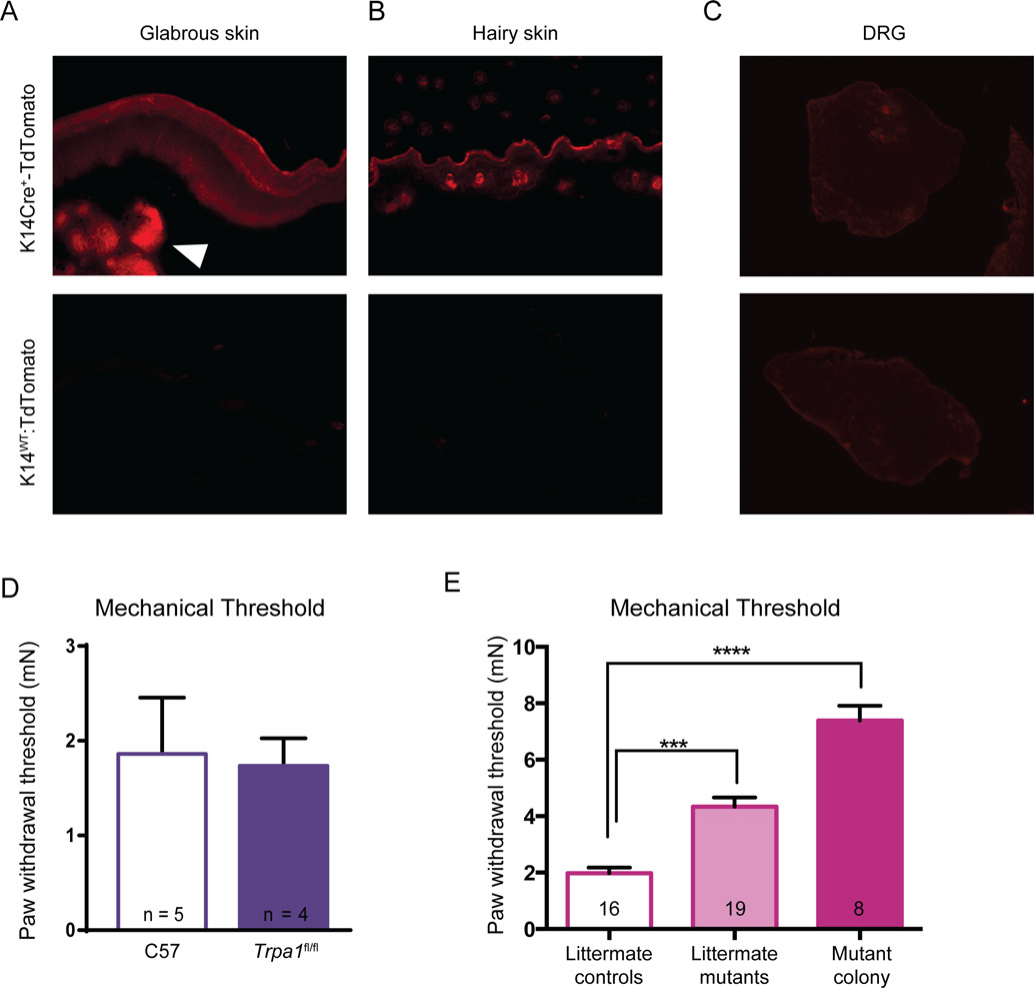
Cre recombinase activity was visualized in epidermal tissues of *K14Cre-tdTomato* reporter mice. (**A-B**) tdTomato reporter fluorescence was observed in the epidermis of both glabrous and hairy skin sections, as expected. In glabrous skin, reporter fluorescence was also observed in sebaceous glands (arrowhead). Bottom row presents the lack of fluorescence in the *tdTomato*^LSL^ mice in the absence of the *K14Cre* allele. (**C**) No tdTomato reporter fluorescence was detected in the DRG of either control or mutant animals, suggesting no ectopic Cre recombinase activity was present in these sensory neurons. (**D**) Importantly, in the absence of the *K14Cre* allele, the *Trpa1*^fl/fl^ mice did not show any mechanical sensory deficit compared to wildtype C57BL/6 mice. (**E**) Mutant animals from the same litters as controls exhibited significant elevated mechanical thresholds (littermate mutants vs. littermate controls). Furthermore, although there was a trend for the mutant animals from the independent colony to have an even greater mechanosensitivity deficit, their mechanical thresholds were not statistically different from those of littermate mutant animals (*p* = 0.0503). Analysis was performed via Kruskall-Wallis and a Dunn’s *post hoc* test. *** *P*< 0.001, **** *P*< 0.0001.

In spite of the strong behavioral and electrophysiological phenotypes observed, the lack of detectable *Trpa1* mRNA expression in epidermis strongly suggested that the phenotype might not be directly caused by to the selective knockout of functional *Trpa1* from keratinocytes. Consequently, alternative mechanisms had to be explored. We first sought to ensure that the mutant mice did not have ectopic, unexpected localization of Cre recombinase activity. Tissue samples from *K14Cre*-*tdTomato* reporter mice were obtained and imaged. tdTomato expression was clearly observed in the keratinocytes of the epidermis of both hairy and glabrous skin (**Fig 7A and 7B**), and also appeared in structures that appeared to be sebaceous glands in the glabrous skin. Others have previously observed K14-mediated expression in other epithelial tissues, as well as the thymus and liver of embryos [22]. No tdTomato expression was observed in the DRG (**Fig 7C**), saphenous nerve, or brain slices from these mice (data not shown), suggesting that Cre activity was restricted to expected tissues, and thus would not have mediated TRPA1 disruption in the nervous system.

We also addressed whether the loxP sites inserted into the introns of the *Trpa1* sequence had an impact on behavioral sensitivity even in the absence of Cre activity. *Trpa1*^fl/fl^ animals had similar behavioral mechanical thresholds as naïve C57BL/6 mice, suggesting the loxP sites had no intrinsic ability to disrupt *Trpa1* mRNA or channel formation (**Fig 7D**). It was also important to consider whether insertion of loxP sites flanking exons 22–24 of the *Trpa1* locus may have unintentionally altered the sequence of another gene on the opposite strand of DNA. Therefore, Cre-mediated recombination within K14 expressing cells could have altered an important functional non-coding DNA element within those cells. However, there are no annotated genes on the opposite strand in this location in mouse (chromosome 1qA3); likewise, there are no known promoters, enhancer regions, miRNAs, or lncRNAs located in this immediate region, as annotated in the UCSC genome browser using the 2011 annotation of the mouse genome (mm10) [23]. However, this search cannot exclude the possibility that such genetic components exist but have not yet been annotated.

As a final control, we reconstituted additional *K14Cre-Trpa1*^fl/fl^ animals such that littermate controls (*K14Cre-Trpa1*^+/+^) would be available for direct comparison in an effort to control as much background genetic variation as possible. These new *K14Cre-Trpa1*^fl/fl^ animals should have the same Cre recombinase activity and deletion of exons 22–24 of the DNA encoding *Trpa1* as in the original mutant mice described above. When the littermate control mice were compared behaviorally to age-matched littermate mutant *K14Cre-Trpa1*^fl/fl^ mice, we were able to replicate that there was a significant impairment in behavioral mechanical sensitivity in the littermate mutant *K14Cre-Trpa1*^fl/fl^ mice (**Fig 7E**).

Since the body of evidence describing the mechanosensitivity deficit could not be linked to the deletion of *Trpa1* in keratinocytes or in other sensory tissues, the diminished mechanosensation in these mice required further exploration. Four possible candidate tissue sites could be responsible for the observed deficit in mechanosensation in the mutant mice: skin ultrastructure or skin function, direct or indirect effects from the immune system, primary sensory neurons, or the central nervous system.

### Skin Structure, Function and Inflammatory Infiltration

To examine skin and subcutaneous structure of the paw, we first performed hematoxylin and eosin staining on hindpaw cross-sections from naïve control and mutant animals to identify any gross morphological differences. The mouse cohorts were indistinguishable from one another in morphology and architecture of the skin, subcutaneous muscle tissue, and metatarsal bones (**S2 Fig**). We next asked whether the overall inflammatory and immune cell response following CFA injection was impaired in these mutant mice. Keratinocytes mediate much of the immune response in the skin, in part through the release of pro-inflammatory mediators such as interleukin-6 (IL-6) or interleukin 1β (IL-1β), which can mediate mechanical hyperalgesia [24–26]. We assessed the stained hindpaw cross-sections of mice 2 days after injection of CFA, and noted a similar degree and distribution of inflammation between genotypes, with each genotype exhibiting an extension of inflammation from the injection site in the plantar area, around the periphery of the paw, and also involving the dorsal subcutaneous tissue. Throughout the subcutaneous space of the injected hindpaws, there was a mixed inflammatory infiltrate comprised of macrophages, neutrophils, and lymphocytes, consistent with the expected inflammatory response to this type of injury (**S3 Fig**). Importantly, there was no apparent difference in cellular constituents of the inflammatory response or level of edema when comparing cross sections of control and mutant hindpaws, despite total paw thickness being slightly reduced in CFA mice following inflammation (**Fig 6C**). Thus, the difference in behavioral responses after inflammation does not appear to be mediated by either skin ultrastructure or recruitment of cellular inflammatory mediators.

### Impaired Signaling between Keratinocytes and Sensory Neurons

Since there was a notable phenotype at the skin-nerve level, we next operated under the assumption that there was either decreased keratinocyte signaling to sensory neurons, or decreased excitability of sensory neurons. We also questioned whether there may be additional phenotypic deficits at the level of the keratinocyte, since this was the site of Cre-mediated recombination, and perhaps an off-target effect may have impacted the function of this cell type.

**Fig 8:**
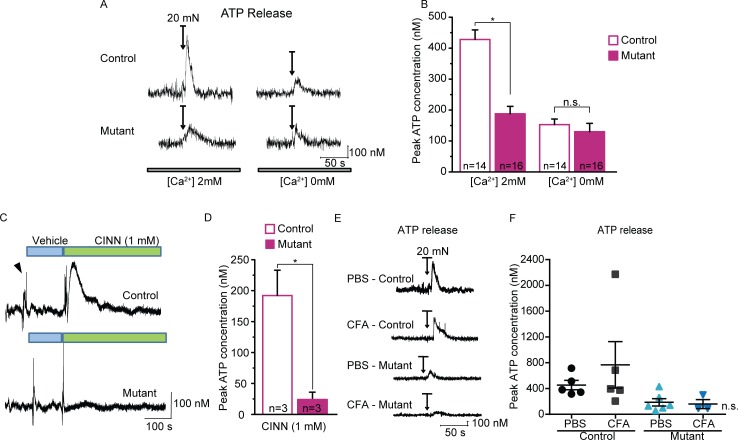
Decreased mechanically- and chemically-evoked ATP release in mutant glabrous skin. (**A**) Example traces of ATP release transient measured from control (top) and mutant (bottom) glabrous skin in response to a 10s, 20 mN mechanical stimulation (applied when indicated by arrows). (**B**) Peak ATP measurements were markedly decreased from the mutant skin compared to control skin. ATP responses from both control and mutant skin were greatly reduced when performed using a calcium-free extracellular solution. (**C**) Sample ATP release traces in response to application of 1 mM cinnamaldehyde (CINN; indicated by green bars). (**D**) Chemically-induced ATP release was greatly reduced in glabrous skin of mutant animals. (**E**) Examples of ATP recordings from control and mutant glabrous skin excised from mice treated with either PBS or CFA. (**F**) Maximal ATP release was not significantly different between PBS and CFA-treated animals of either genotype; a single animal treated with CFA did show a substantially elevated peak ATP response. * *P*<0.05. Data reported as mean ± s.e.m.

We first considered chemical signaling from keratinocytes. ATP has emerged as a leading signaling molecule released from cultured keratinocytes in response to heat, and can activate purinergic ion channels (P2X3, P2X2/3) expressed on sensory terminals [9,27,28]. Further, ATP is released from isolated keratinocytes in response to mechanical stimuli [1,6]. Therefore, we measured mechanically-evoked ATP release from keratinocytes as punctate force was applied to isolated glabrous skin. An enzyme-coated probe was inserted in the skin of control mice to record ATP release *in situ* as a 20 mN von Frey filament was applied to the skin for 10 seconds. The ATP concentration transiently peaked during the mechanical stimulation and continued to decay after force removal (**Fig 8A**). These data show for the first time that ATP release is rapidly evoked by force applied to the skin.

Next, we asked whether the mutant mice have altered ATP releasing capabilities in response to mechanical probing. Interestingly, we found that the peak force-evoked ATP release was substantially reduced by over half (55%) in skin from mutant mice compared to controls (**Fig 8B**, left). We hypothesized that ATP release is likely to be calcium-dependent, since activation of a mechanosensitive ion channel in keratinocytes likely triggers this process. We found that mechanically-evoked ATP release in control skin was reduced by 65% in the absence of extracellular Ca^2+^ compared to a physiological level of extracellular calcium (2 mM), and this reduced ATP release in control skin was at a level comparable to that in the mutant skin (**Fig 8A and 8B**, right). These results suggest that a calcium-permeable ion channel may be important for triggering or mediating ATP release from whole skin.

We next asked whether the ATP release was specific to mechanical stimulation, or whether it could be elicited by cinnamaldehyde, a chemical agonist of TRPA1. Cinnamaldehyde applied directly to the skin evoked a large, extended release of ATP from control skin that was virtually absent in mutant skin (**Fig 8C and 8D**). Given that little to no *Trpa1* mRNA expression was identified in keratinocytes, this response to cinnamaldehyde might not to be due to activation of keratinocytes, but rather is likely an indirect effect through activation of sensory neurons, or even fibroblasts. This finding suggests that the skin from the mutant animals may have a more generalized deficit in releasing ATP, regardless of the source or modality of stimulation.

Finally, we quantified mechanically-evoked ATP release after inflammation. Two days after CFA, there was a trend toward increased mechanically-evoked ATP release from control skin compared to non-inflamed skin. However, this trend was not significant, and was mediated by a single outlier with an exceptionally high peak ATP release. Mechanically-evoked ATP release from inflamed mutant skin was unchanged from saline-injected mutants (**Fig 8E and 8F**).

### Sensory Neurons from Mutant Mice Have Only Subtle Deficits in Chemical Responsiveness

**Fig 9:**
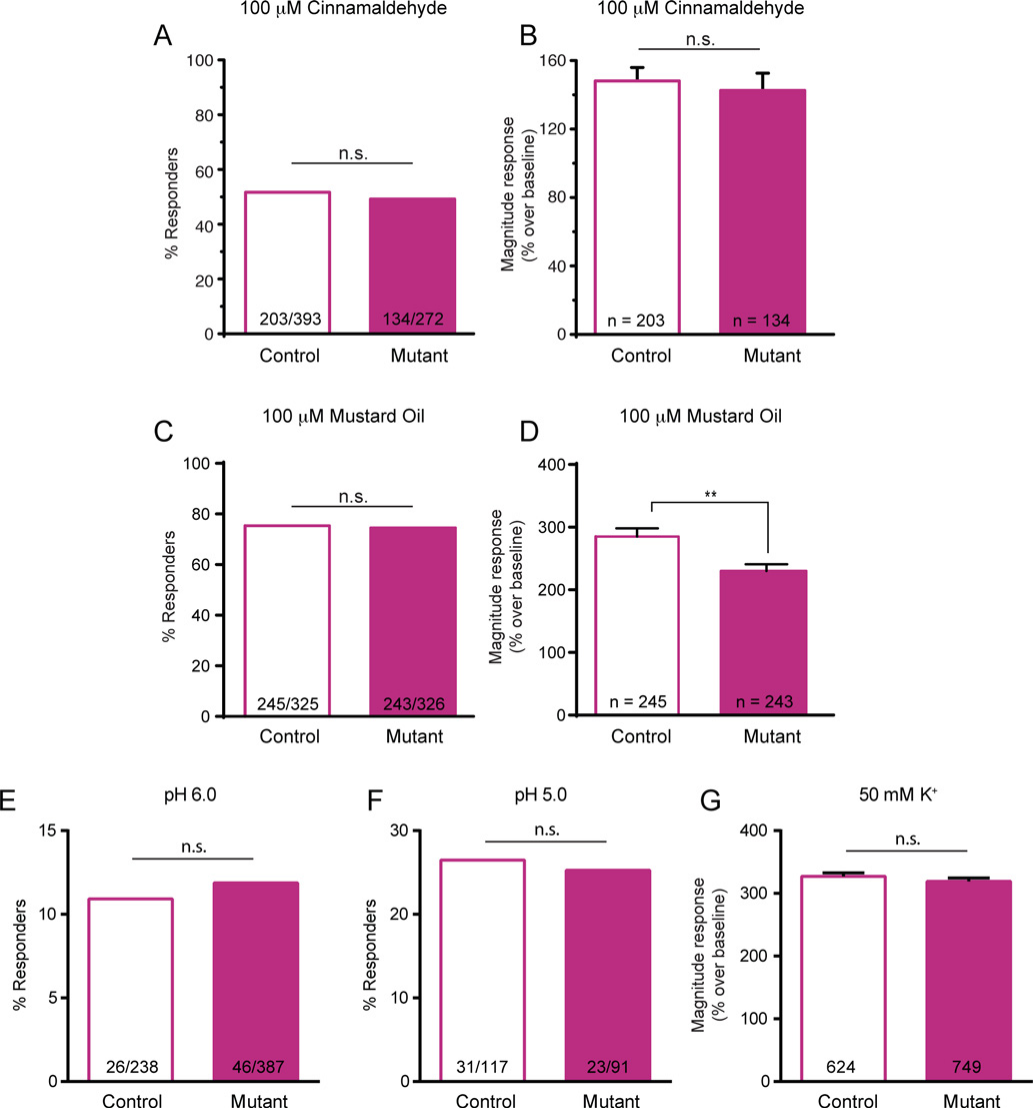
Small-diameter sensory neurons from mutant mice have relatively few deficits in chemical activation. (**A**) Calcium imaging of small-diameter DRG neurons revealed no difference in the percentage of neurons responding to the 100 μM of the TRPA1 agonist, cinnamaldehyde, when comparing control and mutant neurons. (**B**) Magnitude response to cinnamaldehyde was similar between naïve control and mutant small-diameter neurons. (**C-D**) A similar proportion of control and mutant small-diameter neurons responded to another TRPA1 agonist, mustard oil (100 μM; 3 mice per genotype). However, there was a subtle decrease in the magnitude calcium response in mutant compared to control neurons. (**E-F**) Control and mutant sensory neurons responded similarly to application of acidic solutions of pH 6.0 (E) or pH 5.0 (F). (**G**) Combining across multiple experiments, the magnitude response to a depolarizing stimulus of 50 mM K^+^ was similar between control and mutant small-diameter neurons. ** *P*<0.01.

As a second possibility, we sought to evaluate whether the mutant mice may have a generalized deficit in sensory neuron excitability or function. To address this, we tested the responsiveness of isolated small-diameter sensory neurons, which include somata of many C fiber type neurons, to chemical stimuli. A similar percentage of small-diameter (<27 μm) neurons from mutant and control mice responded to the TRPA1 agonist cinnamaldehyde (**Fig 9A**). Control and mutant small-diameter neurons also had a similar magnitude calcium increase in response to 100 μM cinnamaldehyde (**Fig 9B**).

A similar proportion of naïve control and mutant neurons also responded to an additional TRPA1 agonist, mustard oil (**Fig 9C**). These data suggest that a normal number of C fiber type sensory neurons from mutant mice express functioning TRPA1 channels, as would be expected. However, neurons from the mutant mice exhibited a slight deficit in the calcium increase in response to 100 μM mustard oil, with a response 19% smaller than controls (**Fig 9D**). To further assess the excitability of the sensory neurons from these mice, we next stimulated the neurons with an acidified buffer. First, cells were exposed to an extracellular buffer at a pH of 6.0, which caused a significant calcium influx in approximately 10% of the small-diameter neurons from both mouse lines (**Fig 9E**). Additionally, roughly 25% of neurons from both control and mutant mice responded to a pH 5.0 stimulus (**Fig 9F**). In each of these experiments, exposure to 50 mM K^+^ was used to confirm neuronal viability. The calcium responses to potassium-induced depolarization were averaged across all experiments, and indicated that the small-diameter neurons from mutant animals responded with a similar magnitude response as control neurons (**Fig 9G**). Overall, these data suggest that the sensory neurons of naïve mutant animals do not have a global, generalized defect in neuronal activation.

### Gene Expression Profiles Identify Several Differences between Control and Mutant Mice

**Fig 10:**
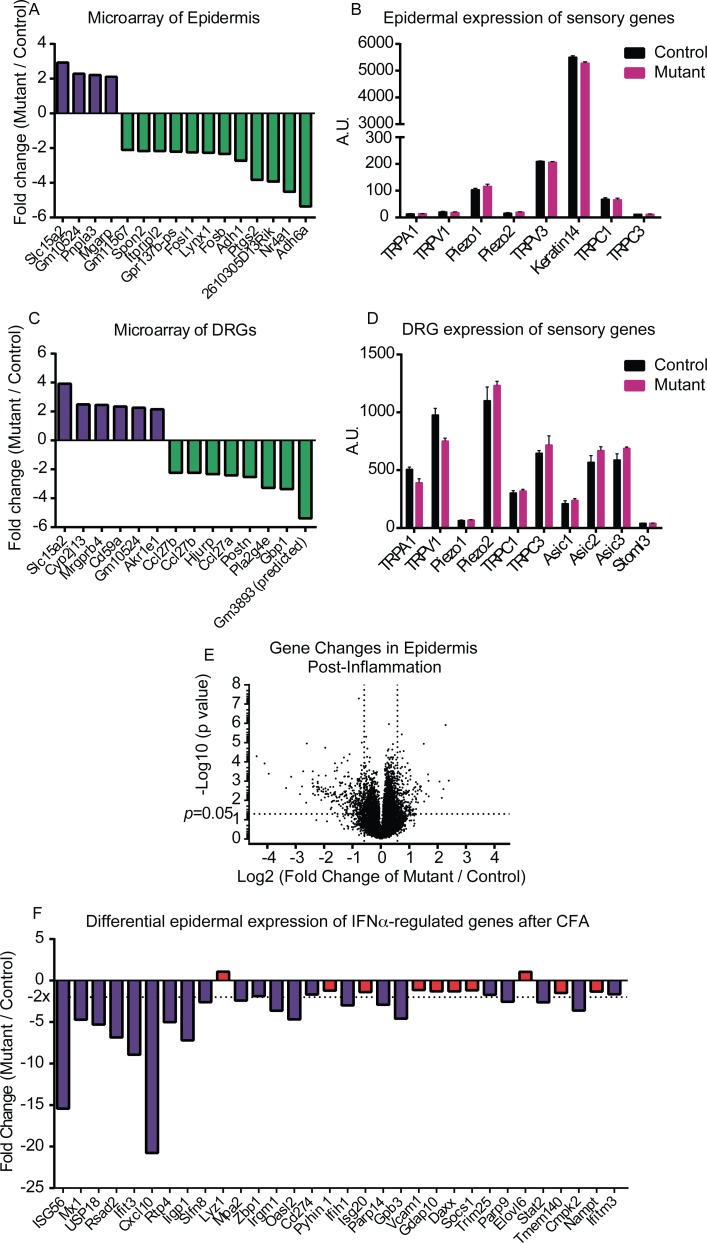
Microarray analysis of control and mutant epidermal and DRG tissues. RNA was isolated from epidermis and DRGs of both control and mutant mice; three biological replicates were included per genotype and treatment group. (**A**) Few genes were dysregulated between mutant and control epidermis in naïve animals. (**B**) Key genes related to sensory function were similarly expressed in control and mutant epidermis. A.U. denotes arbitrary unit. (**C**) Only a small number of genes were differentially regulated in mutant and control DRGs. (**D**) Genes linked to sensory function were expressed at similar levels in control and mutant DRG samples. (**E**) CFA-induced inflammation revealed a much broader set of expression differences between control and mutant epidermis. (**F**) A set of genes regulated by IFNα was explored; many of these genes appeared to be downregulated in the mutant epidermis compared to control.

Thus far, our cumulative evidence suggests that the deficit leading to diminished mechanosensation is occurring at the level of the skin, with only a mild alteration in sensory neuron function. We continued searching for a mechanistic cause for the phenotype by assessing gene expression levels in both the epidermis and the peripheral sensory neurons. We performed microarray analyses on both isolated epidermis and DRGs from the control and mutant mice. mRNA was first extracted from the epidermis of naïve mice of both strains, and also from epidermis of mice of both genotypes two days after induction of inflammation with CFA. In naïve animals, there were only a handful of differences observed between control and mutant mice. Due to the limited changes, this initial analysis was done in the absence of an FDR (false discovery rate) correction. Of all the genes tested, only 16 genes had a significant difference and a fold change greater than two-fold. Of this gene list, *Slc15a2*, *Pnpla3*, and *Mgarp* were significantly higher in the mutant epidermis, and *Adh6a*, *Adh1*, *Nr4a1*, *Ptgs2* (COX-2), *Lynx1*, and *Itpripl2* were significantly lower in the mutant epidermis (**Fig 10A**). Genes known to be involved in sensation and potentially expressed in epidermal tissues were not differentially regulated between the mutant and control epidermis (**Fig 10B**).

Additional microarrays were performed on mRNA isolated from whole DRGs of the animals. Unsurprisingly, there were very few changes in gene expression between the control and mutant mice; five genes were upregulated in the mutant, and five downregulated (**Fig 10C**). Key genes known to be important for mechanosensation, thermosensation, and pain were not different between genotypes (**Fig 10D**).

Prompted by the limited changes observed at baseline, we also assessed the epidermal gene expression profiles 2 days after hindpaw CFA inflammation. Following CFA, a host of other gene expression changes were observed, with 24 genes upregulated and 106 genes significantly downregulated in mutant epidermis as compared to controls (**Fig 10E**). As expected, a great number of inflammation-related genes were induced by CFA in both the control and mutant animals. Perhaps more interestingly, there was a distinct difference in the response to inflammation between the control and mutant epidermis. It appeared that the signature of genes induced by interferon alpha (IFNα) was not induced as well in the mutant animal epidermis. For example, of a list of 32 genes induced by IFNα in CD8^+^ T cells [29], 22 of these were significantly lower in expression in the mutant animals compared to control animals following inflammation (**Fig 10F**). More specifically, of the 10 IFNα-regulated genes most strongly induced, 9 were significantly downregulated in the mutant epidermis.

## Discussion

We set out to determine the functional contribution of TRPA1 in keratinocytes by generating *K14Cre-Trpa1*^fl/fl^ mutant mice that lack *Trpa1* expression in keratinocytes. After extensive searching, we observed remarkably little *Trpa1* expression in the epidermis, as it appears either absent or below the limit of detection. As such we were unable to document any reduction of *Trpa1* in keratinocytes of the mutant *K14Cre-Trpa1*^fl/fl^ mice. Despite this lack of quantifiable knockout of *Trpa1* mRNA from mouse epidermis, the mutant mice exhibited a prominent deficit in mechanical responsiveness at both the behavioral and primary afferent levels in both normal and inflamed conditions. Moreover, the mutant mice exhibited a marked deficit in mechanically-evoked ATP release from the skin both normally and after inflammation.

We fully expected that the *K14Cre*-*Trpa1*^fl/fl^ mice would effectively constitute a keratinocyte-selective conditional knockout of functional TRPA1. The *K14Cre* line has been previously utilized and validated [18], and is publicly available through Jackson laboratories (Stock number 004782). Importantly, keratinocyte-specific activity of Cre appeared consistent with expected localization to epidermal tissues, as indicated by the *tdTomato* reporter experiments. Likewise, we observed that the *Trpa1*^fl/fl^ mouse mated with an Advillin-Cre line created an efficient conditional knockout in peripheral sensory neurons (data not shown). For these reasons, it is expected that the *K14Cre*-*Trpa1*^fl/fl^ mice would have the intended genetic modification only in keratin 14-expressing cells. Further, we sought to ensure that K14-expressing cells are not simply expressing an isoform of *Trpa1* undetected by the primer sets used. There is indeed evidence of multiple splice variants of *Trpa1*. For example, *Drosophila* express at least four different splice variants, and the isoforms differ in their thermosensory capability [30]. In mouse, an isoform of *Trpa1* lacking exon 20 has been implicated in the regulation of *Trpa1* expression [31], and other currently unidentified isoforms of *Trpa1* might occur in mouse epidermis. To address this possibility, we used multiple primer sets targeting multiple regions and exons of the *Trpa1* transcript. None of these primer sets revealed detectable *Trpa1* transcript in mouse keratinocytes.

Since *Trpa1* was not detected in adult keratinocytes, it is possible that disruption of *Trpa1* expression in K14-expressing cells at some time during development may play a regulatory role in determining adult mechanical sensitivity. To this effect, we did not detect *Trpa1* expression in K14-expressing (tdTomato+) cells assessed at different stages of development between E10.5 and E16.5. Importantly, these samples would have included cells destined to become both keratinocyte and some Merkel cells, a proportion of which can express K14 [32,33]. Keeping potential species differences in mind, it has been shown that hamster Merkel cells express *Trpa1* [34]. Additionally, *Trpa1* might be transiently expressed in a particular niche of K14-expressing cells later in embryonic development. Merkel cell development and maturation is a process involving expansion within touch domes between E16.5 and E18.5, and also continues throughout development into the perinatal timeframe [35,36]. As such, we cannot exclude the possibility that transient and restricted expression of *Trpa1* may lead to developmental impacts on mechanosensation.

Overall, our data suggest that the deficits in mechanosensory behavior and suprathreshold afferent firing in the mutant mouse are therefore likely due to factors other than *Trpa1* deletion from keratinocytes, given the lack of its expression in control keratinocytes. It is unlikely that the presence of only a few, sparse transcripts of *Trpa1* in the epidermis of an entire paw would be responsible for a large proportion of an organism’s mechanical responsiveness. Instead, the observed phenotypes might be related to impaired ATP release from the mutant skin.

### Site(s) of Deficit in Mutant Animals—Skin, Peripheral Sensory Neurons or Central Nervous System (CNS)

We proposed that the marked phenotype observed in the mutants could be due to a deficit at one of three locations: the skin, peripheral sensory neurons or central neurons within the spinal cord or higher. First, the phenotype could stem from the initial site of contact between an external sensory stimulus and the body: the skin. We found no gross morphological changes in the epidermis or dermis, indicating that differences in skin thickness or structure do not likely underlie the functional changes observed in sensory afferents. There may, however, be changes in the ability of skin cells to release factors that signal to other skin cells or to sensory neurons, such as ATP [6,37,38]. Our data indicate that ATP release in response to mechanical stimulation of the skin is strongly diminished in mutant animals. The source of ATP could be keratinocytes, sensory neurons, or other cell types in the skin such as Merkel cells, or potentially even dermal cells including fibroblasts. Though our current technique does not isolate ATP release from different cell types, keratinocytes vastly outnumber other skin cells present in the epidermis, and documented evidence from isolated keratinocytes shows they are capable of releasing ATP [1,6,9]. This ATP release may be either vesicular or non-vesicular [1]. Therefore, the decreased ATP release in the mutant skin could be due to decreased ATP production, packaging, or release. No known candidate genes involved in ATP release were identified as dysregulated within the epidermis in the microarray studies.

Just as the mutant skin exhibited decreased ATP release, there could be a parallel deficit in release of other factors that may signal from keratinocytes to sensory neurons. Additional postulated signaling factors include β-endorphin, endothelin-1, neurotrophins, and multiple interleukins [39]. There remain multiple possibilities through which these and other mediators may activate or sensitize sensory neurons.

We subsequently assessed the gene expression profiles of the epidermis from these mice. There were a minimal number of genes differentially regulated in the epidermis, with only 4 upregulated and 12 downregulated in mutant uninflamed tissues compared to controls. These genes have largely not been previously linked to mechanosensation, pain, or ATP release. One exception is the observed downregulation of *Ptgs2*, the gene that encodes for cyclooxygenase-2, in mutant epidermis. This enzyme acts on arachidonic acid in the prostaglandin synthesis pathway, and is a target for the commonly-used non-steroidal anti-inflammatory compounds [40,41]. Since these drugs inhibit the activity of cyclooxygenase-2 to decrease pain, it follows that decreased expression of *Ptgs2* could lead to dampened mechanosensation. On the other hand, we would not expect much activation of the *Ptgs2*/cyclooxygenase-2 pathway in uninflamed animals, and therefore this might not be the causative factor for the diminished sensation of the naïve mutant mice. It remains a possibility that any of these genes may indirectly be impacting the function of additional genes or proteins responsible for sensory processing.

A second possibility is that the sensory neurons themselves are the source of the mechanosensory deficit. Specifically, our data indicate that primary afferent neurons *in situ* innervating the skin exhibit decreased suprathreshold firing in the mutant animals. By nature, these recordings can be impacted by changes in the skin cells and/or the sensory neurons. These neurons showed no changes in conduction velocity or von Frey threshold; however, more subtle defects could be present within the sensory neurons. Furthermore, our data indicate that a normal percentage of sensory neurons from mutant mice responded to the TRPA1 agonists cinnamaldehyde and mustard oil, suggesting that TRPA1 remains functionally expressed within sensory neurons. Mutant neurons had a normal magnitude response to cinnamaldehyde and to a depolarizing potassium stimulus, though this was in contrast to the subtle deficit in calcium influx in response to mustard oil. One possibility for the subtle (less than 20%) deficit in response to mustard oil is a non-TRPA1-mediated effect of mustard oil. It is also possible that chronically decreased ATP release from the skin could tonically alter the excitability of the sensory neurons. Gene expression profiles in DRG did not support any broad changes that readily explain this deficit, nor did they provide sufficient mechanism to identify the cause of decreased afferent sensitivity. Overall, the sensory neurons from both control and mutant mice appeared quite similar.

The spinal cord, brainstem, or higher levels of the nervous system together compose a third site that could underlie the difference in behavioral responses observed. CNS modulation could retrogradely affect sensory terminals or alter descending modulation of spinal cord reflexes. Although we cannot exclude these possibilities, two observations suggest otherwise: first, the peripherally-restricted and isolated skin-nerve preparations exhibited reduced mechanical firing of primary afferent neurons, and second, the mechanical probing of isolated skin resulted in decreased evoked ATP release in the complete absence of the CNS. Taken together, these data suggest that the most likely site of the deficit is at the level of communication between skin cells and peripheral sensory terminals.

### Peripheral Inflammation in Mutants

The mutant animals also exhibited a blunted response to CFA inflammation in the hindpaw in the form of diminished behavioral sensitization. One possibility is that the adaptive immune response to CFA may be muted in these mice. However, we found no apparent evidence for reduced recruitment of inflammatory cells or specific immune cell types in the skin following CFA, no difference in the level of localized inflammatory infiltrate, and only a slight reduction in edema as measured by total paw thickness. Thus, there was no obvious gross deficit in the immune response to CFA.

Following inflammation, it was readily apparent that localized inflammation induced a strong transcriptional response in the skin. This effect was largely retained in the mutant epidermis; however, a diminished response of the IFNα-regulated genes stood out. Many of the INFα-mediated gene transcription effects are mediated by the transcription factor Stat1 [42]. Interestingly, *Stat1* mRNA was downregulated in the control mice 11-fold after CFA; in the mutant epidermis, there was no such regulation of *Stat1*. While some of the genes differentially regulated after inflammation are likely being differentially expressed by the control and mutant keratinocytes, some of the genes upregulated by inflammation in both mouse lines are expected to be due to mRNA derived from resident or infiltrating immune cells. That is, although the analysis was performed in the epidermis, the differences between the control and mutant mice may actually be localized to these immune cells. For example, there could be decreased infiltration of a specific immune cell population, or simply a modified cytokine profile released from the present cells. Many inflammatory mediators released are known to act both indirectly and directly on sensory neurons, leading to hypersensitivity of inflammation [43–45]. Therefore, there are many possible subcellular defects in the inflammatory response that could partly explain the dampened mechanical allodynia after CFA in the mutant mice.

### *Trpa1* Is Not Detected in Mouse Keratinocytes

Prior studies, including those from our lab, have shown TRPA1 expression in the mouse skin. We previously showed that a TRPA1 antibody labels mouse epidermis, and that labeling is reduced in a global TRPA1 knockout [13]. However, here we were unable to consistently demonstrate that *Trpa1* is present in mouse skin at the mRNA level using multiple qPCR primers, sensitive digital droplet PCR, qPCR on isolated K14-expressing keratinocytes. Despite our lack of reliable expression detection, it remains possible that an exceedingly low copy number of *Trpa1* mRNA transcripts may be found in mouse epidermis, potentially from cells other than keratinocytes themselves. Alternatively, in theory one could pick up a stray *Trpa1* transcript that had been transported to a sensory neuron’s terminals innervating the skin, or a rare transcript from keratinocytes at such low amounts of transcript copies per cell that expression is largely below the limit of detection. However, the physiological relevance and impact of such few transcripts remains debatable. Overall, these results provide no evidence of *Trpa1* expression within mouse keratinocytes. Previous findings suggesting TRPA1 expression in epidermis may now be interpreted with the following caveats: 1) antibody staining for membrane-bound proteins (particularly TRP channels) is difficult and may be non-specific, and 2) human skin may indeed express more *Trpa1* mRNA than mouse skin, as species and body region differences may occur [15]. Further, our findings are in accordance with a recent study in which *Trpa1* expression was not detected in the neck skin of mice [16]. In all, normal mouse epidermis from the various genetic backgrounds tested in this study does not contain appreciable *Trpa1* mRNA. As such, it may be expected that such limited expression would not be capable modifying the functioning of keratinocytes to significantly mediate the whole animal’s behavioral responsiveness to a mechanical stimulus.

### Separated Maintenance of Genetically-Related Mouse Lines May Unintentionally Confound Observed Phenotypes

As discussed, levels of *Trpa1* mRNA were frequently undetected in the epidermal keratinocytes of either the control or mutant animals. One final concern was that following the initial pairing between the *K14Cre* mice and the *Trpa1*^fl/fl^ mice, the colonies of the control *K14Cre*-*Trpa1*^+/+^ and mutant *K14Cre*-*Trpa1*^fl/fl^ mice were maintained independently for multiple generations. Naturally, it is easiest to detect the function of a gene of interest in transgenic animal studies when the background genetic differences are minimized, as it limits the differences in complex interactions between multiple proteins and genes that could be simultaneously impacting the phenotype being studied. Although this common method of independent colony maintenance can provide significant and important savings in cost and time since it greatly limits the production of unused heterozygous mice, the limitations should be very carefully considered and our study underscores the importance of this. The likelihood of genetic drift occurring in one or both of the independently-maintained mouse lines increases with the length of time the colonies are kept separate. For these reasons, there was concern that the phenotypes observed may have been the result of an acquired background genetic variant. However, additional experiments utilizing mutant animals born from the same litter as controls also revealed impaired mechanosensitivity, suggesting that the sensory deficit may be a result of disruption of the DNA encoding the pore region of *Trpa1* in keratin 14-expressing cells at some point during development. Possible genetic drift is a potentially large confounding factor in perhaps a considerable number of studies, although it is rarely considered in the literature as a source of variation. This can be partly overcome by sporadically interbreeding the colonies (for example, creating littermate control animals). Therefore, it is important to remain critical in evaluating genetic studies, carefully ensuring that the phenotypic changes are truly due to the intended genetic manipulation.

Taken together, we can conclude the following from this study. First, *Trpa1* is at best minimally present in the epidermis of control mice, particularly those on a mixed C57BL/6, CBA, Swiss Webster background as used in these studies. Second, our findings highlight the critically important value of having littermate control animals available wherever possible. Lastly, a decrease in ATP release from the skin is correlated with a decrease in mechanosensation. Since keratinocytes appear to signal to the sensory system, the skin remains a viable site for administration of topical pharmacological therapeutics to target cutaneous pain. Continued efforts to delineate the role of keratinocytes in mechanosensation could uncover a deeper understanding of mechanotransduction and somatosensory biology.

## Methods

### Generation of TRPA1 Conditional Knockout Mice

To generate keratinocyte-specific *Trpa1* conditional knockout animals, loxP sites and a FRT flanked neomycin resistance cassette were placed around exons 22 through 24, which encode for the pore domain of TRPA1, in mouse embryonic stem (ES) cells by homologous recombination (**S1 Fig**). ES cells were selected by culturing in 200 μg/ml of neomycin and screening for the appropriate recombination event by Southern blotting. Chimeric animals were generated from ES cells containing the targeted *Trpa1* conditional knockout allele and mated with Flpe expressing mice to excise the neomycin resistance cassette; this constituted the *Trpa1*^fl/+^ mouse. Congenic *Trpa1*^fl/+^ mice were backcrossed ten times to C57BL/6 animals, then intercrossed to create *Trpa1*^fl/fl^ mice. *Trpa1*^fl/fl^ mice were mated to a *Keratin 14(K14) Cre* recombinase mouse (Jackson laboratories, stock number 004782) [18]. Under the K14 promoter, Cre recombinase is expressed in all keratinocytes, as early as E9.5 [17,18,46]. The resulting mice were *K14Cre*-*Trpa1*^fl/fl^ (termed “mutant” animals). Control animals expressed *K14Cre* and were homozygous for the wildtype allele for *Trpa1* (*K14Cre-Trpa1*^*+/+*^; control mice). We confirmed that epidermal tissues from *K14Cre*-*Trpa1*^fl/fl^ mutant animals contained genomic DNA for the excision product, confirming effective Cre-mediated recombination at the Trpa1 locus (**Fig 1A**). This DNA product of loxP site-directed excision was absent in DRGs from either control or mutant animals (**Fig 1A**). Both control and mutant mice were maintained in independently-generating colonies for many generations to reduce the production of heterozygous mice, which were not used in these experiments. Unless explicitly noted, mutant mice used throughout this manuscript were obtained from the independent colony. Adult male mice of at least 7 weeks of age were used, and were either control or mutant mice on a mixed genetic background including C57BL/6, CBA, and Swiss Webster strains, which had been crossed onto a C57BL/6 background at least once after introduction of the *K14Cre* background, then intercrossed several times. In some experiments, *K14Cre* animals were mated with a *tdTomato* reporter mouse expressing *tdTomato* behind a loxP-flanked STOP cassette (*tdTomato*^LSL^). Mouse genotype was confirmed by PCR. Mice were anesthetized with isoflurane and euthanized by either cervical dislocation or decapitation. Animal use and welfare procedures adhered to the NIH Guide for the Care and Use of Laboratory Animals, and followed protocols approved by the Medical College of Wisconsin Institutional Animal Care and Use Committee (approval #: 383). Experimenters were blinded to mouse genotype throughout all behavioral, electrophysiological and morphological experiments.

### Behavior

All mechanical behavior tests were carried out on the glabrous surface of the hindpaw. Mechanical threshold was determined using calibrated von Frey filaments and the Up-Down method [47,48]. The frequency of withdrawal to a suprathreshold mechanical 3.31 mN stimulus was tested by applying a von Frey filament 10 times to the plantar surface of the hindpaw [27,49]. In the needle test, a spinal needle was applied 5 times to the glabrous skin of the hindpaw and the percentage of responses was recorded. The light touch behavioral assay was used to quantify response to innocuous punctate and dynamic stimuli as previously described [50]. Furthermore, heat sensitivity was quantified by measuring the withdrawal latency to a focal radiant heat source [51]. Cold sensitivity was determined using a static cold plate (0° or 10°C); the withdrawal latency and number of paw responses (lifts, licks, flutters, or holds) were measured during a 5 min test.

### Teased Fiber Skin-Nerve Recordings

The *ex vivo* sural skin-nerve preparation was utilized to determine mechanical response properties of cutaneous primary afferent fibers in control and mutant mice following established protocols [13,52]. Briefly, *ex vivo* skin-nerve preparations were dissected from the glabrous hindpaw and immediately placed into the recording chamber with the corium side down on top of a nylon-covered mesh, allowing perfusion of the skin through the dermis. The recording chamber was superfused with oxygenated synthetic interstitial fluid at 32 ± 0.5°C. We classified units as Aβ when the axon conduction velocity was over 10 m/s, Aδ units as A-mechanoreceptors (AM) for velocities between 1.2–10 m/s with slowly adapting action potential firing, and C fibers for velocities under 1.2 m/s. A feedback-controlled mechanical stimulus was applied for 10 seconds to the receptive field. Action potentials were recorded and analyzed using the data acquisition software LabChart 7 Pro (ADInstruments, Colorado Springs, CO).

### Induction of Peripheral Inflammation Using Complete Freund’s Adjuvant (CFA)

Mice were anesthetized by isoflurane and intraplantar injections of 50 μl undiluted CFA (30μg/μl; Sigma) were given in the proximal plantar hindpaw. Phosphate-buffered saline (pH 7.4) injected at the same volume and location was used as a vehicle control. All CFA behavioral and skin-nerve experiments were performed 2 days after either CFA or vehicle injection.

### Quantification of Skin Thickness

Glabrous forepaw skin was dissected free from mice. Skin sections were transferred to a 10% zinc formalin solution and embedded in paraffin. The tissue was then sliced into 4 μm sections, plated onto glass slides, stained with hematoxylin and eosin, and visualized by light microscopy. Nikon NIS-Elements software was used to manually trace both the epidermis, and epidermis and dermis combined, in order to compute total area within 300 μm sections. Skin thickness was then calculated from the total traced area.

### Quantification of Paw Inflammation

The hindpaws of mice injected 2 days earlier with CFA or PBS were measured for total paw thickness (height). Excised hindpaws were fixed in formalin and sectioned coronally at the mid paw. Fixed tissues were processed at the Children’s Hospital of Wisconsin Research Institute Histology Core Facility. Hindpaw tissue sectioned and stained with hematoxylin and eosin. The character, degree, and distribution of inflammation and tissue damage were assessed by a pathologist (M.W.L.). A Hamamatsu Nanozoomer HT slide scanner (Hamamatsu Photonics, K.K., Hamamatsu City, Japan) was used to generate low-power (1.25x) images of the tissue, and the area of inflammation was manually measured using NDP.View 1.1.5 software at the Children’s Research Institute Imaging Core Facility. High-powered (400x) microscopic images were obtained using an Olympus BX53 microscope and cellSens Standard 1.5 software (Olympus, Inc., Tokyo, Japan).

### Quantification of Extracellular ATP Release

Dual simultaneous amperometric recordings were made with purine biosensors [53] (Sarissa Biomedical Limited, Conventry, England). The ATP biosensor is a platinum microelectrode coated with an ultrathin biolayer containing glycerol kinase and glycerol-3-phosphate oxidase. In the presence of ATP, these enzymes catalyze two sequential reactions leading to production of H_2_O_2_, which is then detected via oxidation of the electrode connected to amperometric recording equipment. The ATP sensor responds rapidly (10–90% rise in <10 sec) and exhibits a linear response to ATP over physiologically-relevant concentrations [54]. A null probe (sarissaprobe Null) lacks the enzymatic biolayer and is used to control for non-specific recordings, for example, electro-active interference, including artifacts from movement, skin stretch, temperature or pH signals [53,55,56]. Biosensors were used in conjunction with a dual-channel DY2021 potentiostat and recording system (Digi-Ivy, Inc., Austin, TX, USA). Enzymatic microelectrodes were calibrated to known ATP concentrations before and after each set of experiments. Biosensor sensitivity was increased by cycling the sensors from -500 mV to +500 mV at a rate of 100 mV/s for 10 cycles [53]. Glabrous skin was excised from the hindpaw as it was for the skin-nerve recordings, above. The sensing portions (0.5 mm in length) of the biosensors were inserted into the skin by using MP-225 motorized micromanipulators (Sutter Instruments). The sensors were polarized to +600 mV relative to an Ag/AgCl potentiostat reference electrode that was also placed into the skin. Recordings from the null probe were subtracted from the ATP probe recordings. Final ATP concentrations measured were calculated from calibration response ratios. The ATP release was rapidly detected in response to mechanical stimulation of the skin with a 20 mN von Frey monofilament.

### Calcium Imaging from DRG Cultures

Calcium imaging was conducted as previously described [57], with minor modifications described here. Briefly, all lumbar dorsal root ganglia (DRG) were isolated bilaterally and plated onto laminin-coated glass coverslips. Neuronal medium consisted of Dulbecco’s modified Eagle’s medium/Ham’s F12 medium, supplemented with 10% heat-inactivated horse serum, 2 mmol/L L-glutamine, 0.5 to 0.8% glucose, 100 units/mL penicillin, and 100 μg/mL streptomycin, with no exogenous growth factors. Calcium imaging was performed using the dual-wavelength fluorescent calcium indicator FURA-2AM (Invitrogen). Once cells were loaded with 2.5 μl/mL FURA-2AM, coverslips were mounted onto a perfusion chamber and superfused with buffer at 6 mL/min. Fluorescence images were captured with a cooled CCD camera (CoolSNAP FX; Photometrics, Tucson, AZ), and in some experiments with a cCMOS camera (Zyla; Andor Technology LTD, Belfast, UK). MetaFluor (Molecular Devices, Sunnyvale, CA) and NIS Elements (Nikon Instruments, Melville, NY) imaging software were utilized in order to detect and analyze intracellular calcium changes throughout the experiment. A ≥20% increase in intracellular calcium from baseline was considered a response to a stimulus. In one set of experiments, neurons were superfused with 100 μM cinnamaldehyde [57] for 3 min and peak increases in Fura-2 ratio from baseline measured. In a separate set of experiments, neurons were superfused with 100 μM mustard oil [57] for 1 min. A final set of experiments measured calcium responses to perfusion with extracellular buffer at pH 6.0 or at pH 5.0. At the end of each protocol, 50 mM potassium chloride solution was superfused for 30 sec to determine neuronal viability of non-responsive cells. Neurons were considered small if the average somata diameter was less than 27 μm and only small-diameter neurons were included in this study in order to concentrate inclusion of C fiber type somata [58]. For experiments utilizing mice that received CFA-inflammation, lumbar DRG at spinal levels L3-L5 ipsilateral to the injection site were isolated for calcium imaging experiments and small-diameter neurons were imaged as described above.

### Gene Expression

Gene expression studies were performed on both epidermis and isolated DRG from mice of varying genotypes, as defined where applicable. Glabrous hindpaw skin was dissected from the mutant and control mouse controls. Skin samples were incubated for 50 minutes in dispase (10 mg/mL), and the epidermis was peeled from the dermis. RNA was isolated from this epidermal layer using TRIzol and the PureLink RNA Micro kit (LifeTechnologies, Carlsbad, CA). Additionally, DRG (T5-L6) were dissected, stored overnight in RNA*later* at -20°C, and RNA isolated using TRIzol Reagent following the manufacturer’s recommendations (LifeTechnologies). Total RNA content was assessed with a NanoDrop Lite spectrophotometer (Thermo Scientific, Wilmington, DE).

cDNA was generated using the SuperScript III First-Strand Synthesis System (Invitrogen, LifeTechnologies), using a combination of both random hexamers and oligo dT to initiate reverse transcription of a varied pool of transcripts. Real-time, quantitative polymerase chain reaction (qPCR) was performed on a Mastercycler ep Realplex^2^ thermal cycler (Eppendorf, Hamburg, Germany). Real-time PCR using Sybr Green was performed using primer sets described in **S1 Table**. Gene expression was normalized to *Gapdh*. Additionally, digital droplet PCR was used since as a secondary, highly-sensitive method useful in performing quantitative PCR on samples where low expression levels of genes are expected. The digital droplet PCR method has been used clinically and is validated for its high sensitivity in detecting low copy number reliably [59].

To increase specificity, we also isolated keratin 14-expressing cells from the skin from both adult and embryonic animals. Skin from *K14Cre-tdTomato* reporter mice were dissected and dissociated into a single cell suspension. Fluorescence-activated cell sorting using a FACSAria (BD Biosciences, San Jose, CA) sorted the tdTomato-expressing cells directly into TRIzol LS (LifeTechnologies), and RNA was isolated from this cell population as described above.

We further aimed to increase qPCR sensitivity on RNA isolated from the epidermis by performing gene-specific cDNA generation of TRPA1 followed by additional real-time qPCR of a region of TRPA1 located within the sequence selectively enhanced during cDNA generation. Primer sequences for this nested qPCR protocol are described in **S1 Table**.

### Microarray Analysis

As in the gene expression studies above, microarrays were also performed on both epidermis from the glabrous hindpaw and on isolated DRG (L1-L6). RNA was isolated from these tissues using the PureLink RNA Micro kit as above, and cDNA was generated using the GeneChip WT Plus Reagent kit (Affymetrix, Santa Clara, CA). Samples were processed for microarray analysis according to the manufacturer’s instructions, and as previously described [60]. Labeled and fragmented cDNA was hybridized to GeneChip Mouse Gene 1.0 ST arrays (Affymetrix). All groups included three biological replicates. Microarray datasets have been deposited to the NCBI GEO database under the accession number GSE72672.

### Data Analysis

Single fiber data was compared between mutant mice and controls and total *n*’s are listed (**S2 Table**). For each fiber type, mechanical threshold was compared using Mann-Whitney *U* test, conduction velocity compared using Student’s (two-tailed) *t* test, and the number of mechanically-evoked action potentials across the force range was compared using repeated measures two-way ANOVA with Bonferroni *post hoc* analysis using Prism 6 software (GraphPad, La Jolla, CA). In behavioral tests, paw withdrawal thresholds were analyzed using a Mann-Whitney *U* test; in CFA experiments, withdrawal thresholds were first analyzed by two-way ANOVA, followed by *post hoc* Mann-Whitney *U* tests with a Bonferroni adjustment. All other behavioral assays used Student’s (two-tailed) *t* tests, for two groups, or an ANOVA where applicable. For calcium imaging data comparing two groups, the percentage of neurons responding was compared using Fisher’s Exact and the magnitude of response was compared using a *t* test; for more than two groups, the percent of responding neurons was analyzed with a Chi square and *post hoc* Fisher’s Exact tests; response magnitude was analyzed using one-way ANOVA with Bonferroni adjustment. ATP analyses and skin thickness comparisons used Student’s (two-tailed) *t* tests. *P*<0.05 was considered to be significant for all comparisons. Array data was analyzed using Genomics Suite 3 (Partek, St. Louis, MO) via ANOVA. Genes were considered significantly regulated if they reached a cutoff of a 2-fold change and *P*<0.05 with FDR. All summarized data are reported as mean ± s.e.m.

## Supporting Information

S1 Fig Production of *Trpa1*^fl/fl^ mice. (**A**) Relevant regions of the targeting vector, the endogenous *Trpa1* locus and the expected conditional knockout *Trpa1* allele. Black boxes denote exons in the depicted *Trpa1* genomic region, and white boxes denote exons 22–24 that encode a region encompassing the S5 and S6 transmembrane domains. Only restriction sites relevant for Southern blot analysis are depicted. Proper homologous recombination of the targeting vector to the *Trpa1* locus results in loxP flanked exon 22–24 and insertion of self-excising neomycin cassette. (**B**) *Trpa1* conditional knockout allele after self-excision of the neomycin cassette and the Cre-mediated excised alleles.
(TIF)

S2 Fig Skin thickness is similar between control and mutant mice. (**A**) Examples of dermal and epidermal skin sections from control and mutant mice. (**B**) Average epidermal and dermal thickness measured across 400 μM sections in control and mutant mice. Data reported as mean ± s.e.m.
(TIF)

S3 Fig Gross morphological evidence of inflammatory response appears normal in mutant and control mice. Cross-sections of PBS-injected (uninjured) or CFA-injected (injured) paws from both control and mutant mice were obtained. In injured tissues, the thin yellow border delineates the large area of inflammation within the CFA-injected paw. As shown, this was present in both the control and mutant paw. As evidenced in the 400x images, both control and mutant mice exhibited a mixed inflammatory infiltrate. The magnified inset identifies these components; lymphocytes (yellow arrow), neutrophils (red arrow), and macrophages (blue arrow) were present.
(TIF)

S1 Table PCR primers used throughout this study. (TIF)

S2 Table Summary of fiber properties. (TIF)
