# Supplementary figures and images for: Mechanosensory and ATP Release Deficits following Keratin14-Cre-Mediated TRPA1 Deletion Despite Absence of TRPA1 in Murine Keratinocytes
Source: PLoS One. 2016 Mar 15;11(3):e0151602. doi: 10.1371/journal.pone.0151602 (PMC4792390; doi:10.1371/journal.pone.0151602)

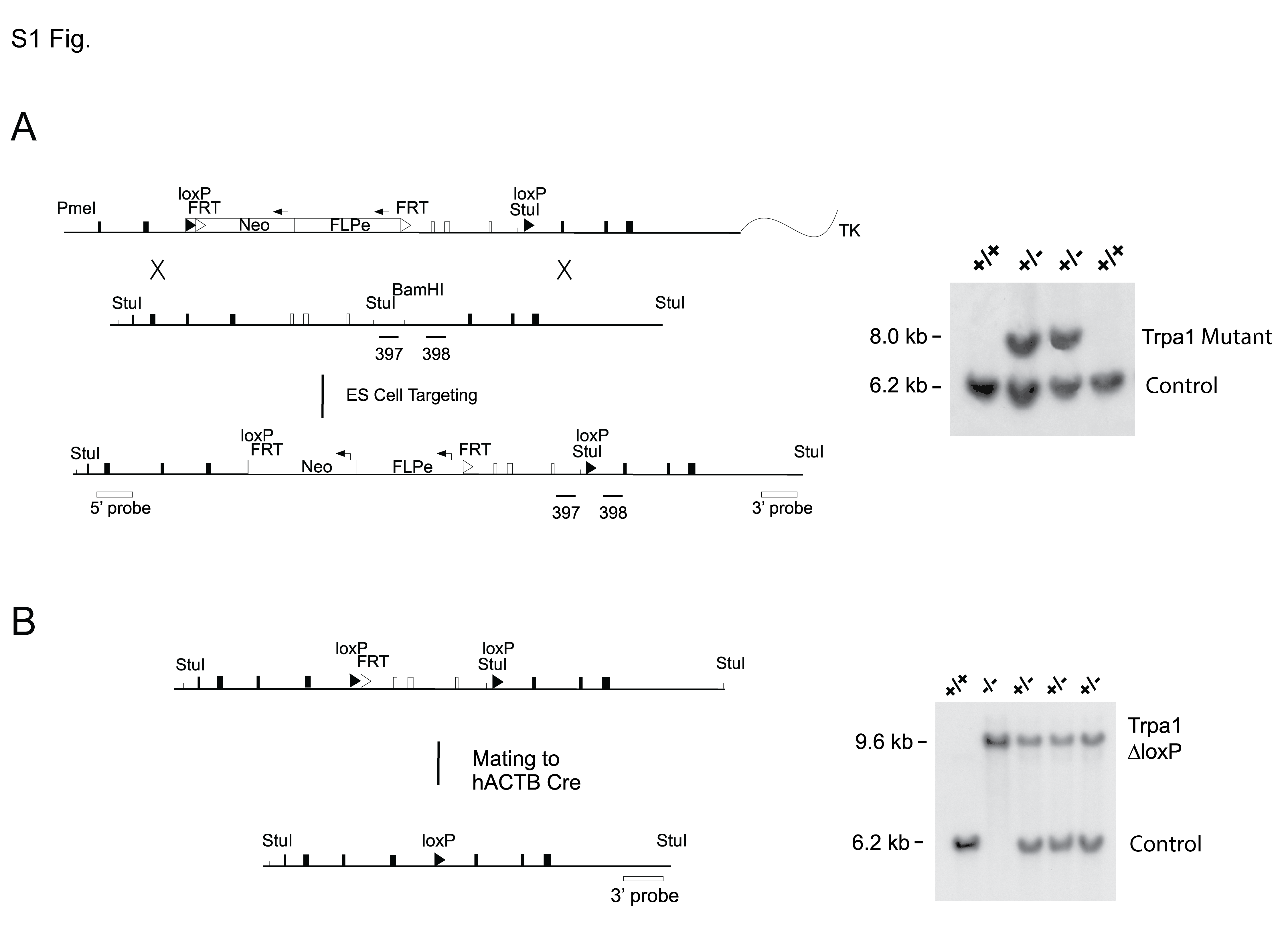

Supplement: S1 Fig — (A) Relevant regions of the targeting vector, the endogenous Trpa1 locus and the expected conditional knockout Trpa1 allele. Black boxes denote exons in the depicted Trpa1 genomic region, and white boxes denote exons 22–24 that encode a region encompassing the S5 and S6 transmembrane domains. Only restriction sites relevant for Southern blot analysis are depicted. Proper homologous recombination of the targeting vector to the Trpa1 locus results in loxP flanked exon 22–24 and insertion of self-excising neomycin cassette. (B) Trpa1 conditional knockout allele after self-excision of the neomycin cassette and the Cre-mediated excised alleles. (TIF) [file pone.0151602.s001.tif]

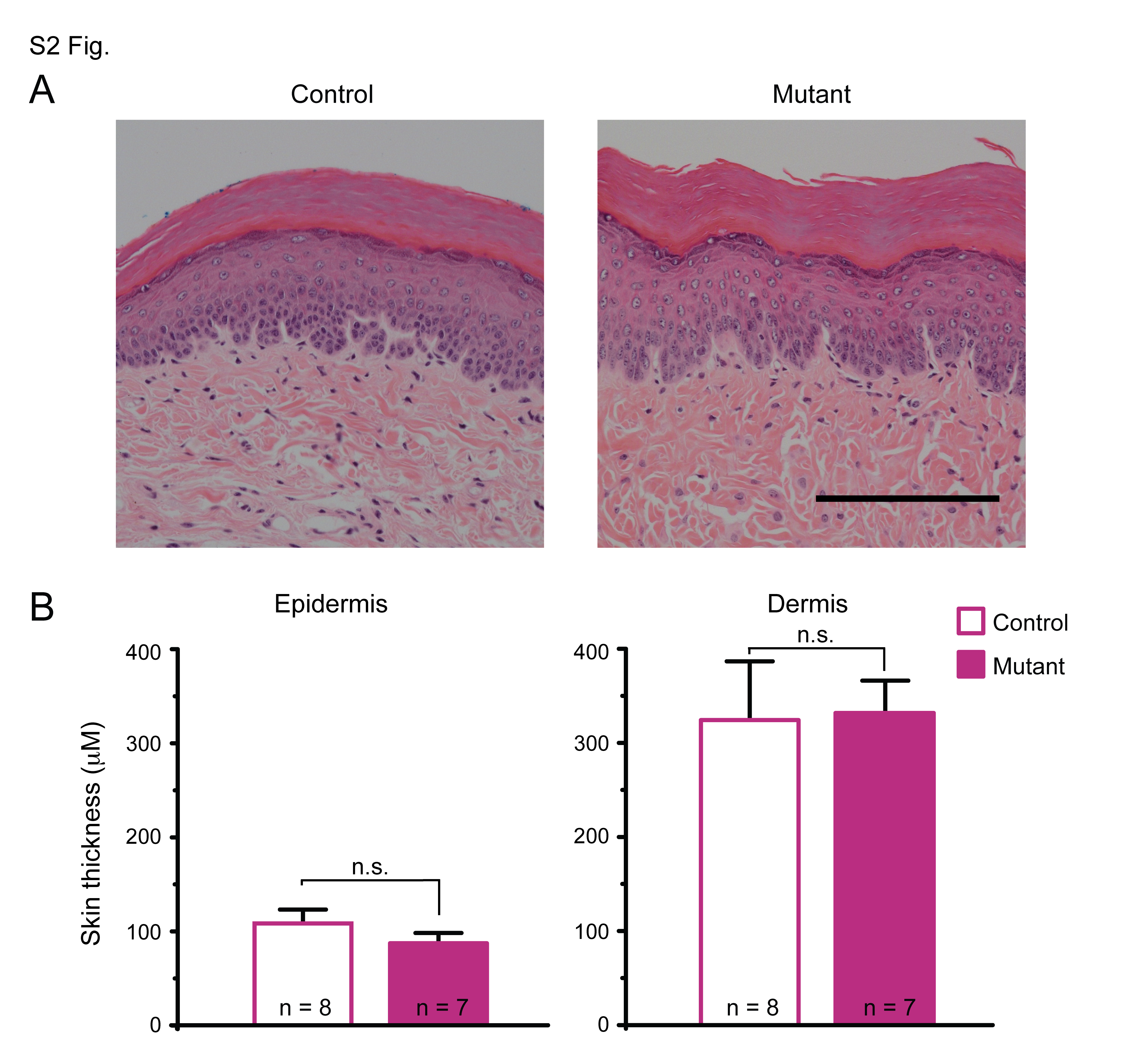

Supplement: S2 Fig — (A) Examples of dermal and epidermal skin sections from control and mutant mice. (B) Average epidermal and dermal thickness measured across 400 μM sections in control and mutant mice. Data reported as mean ± s.e.m. (TIF) [file pone.0151602.s002.tif]

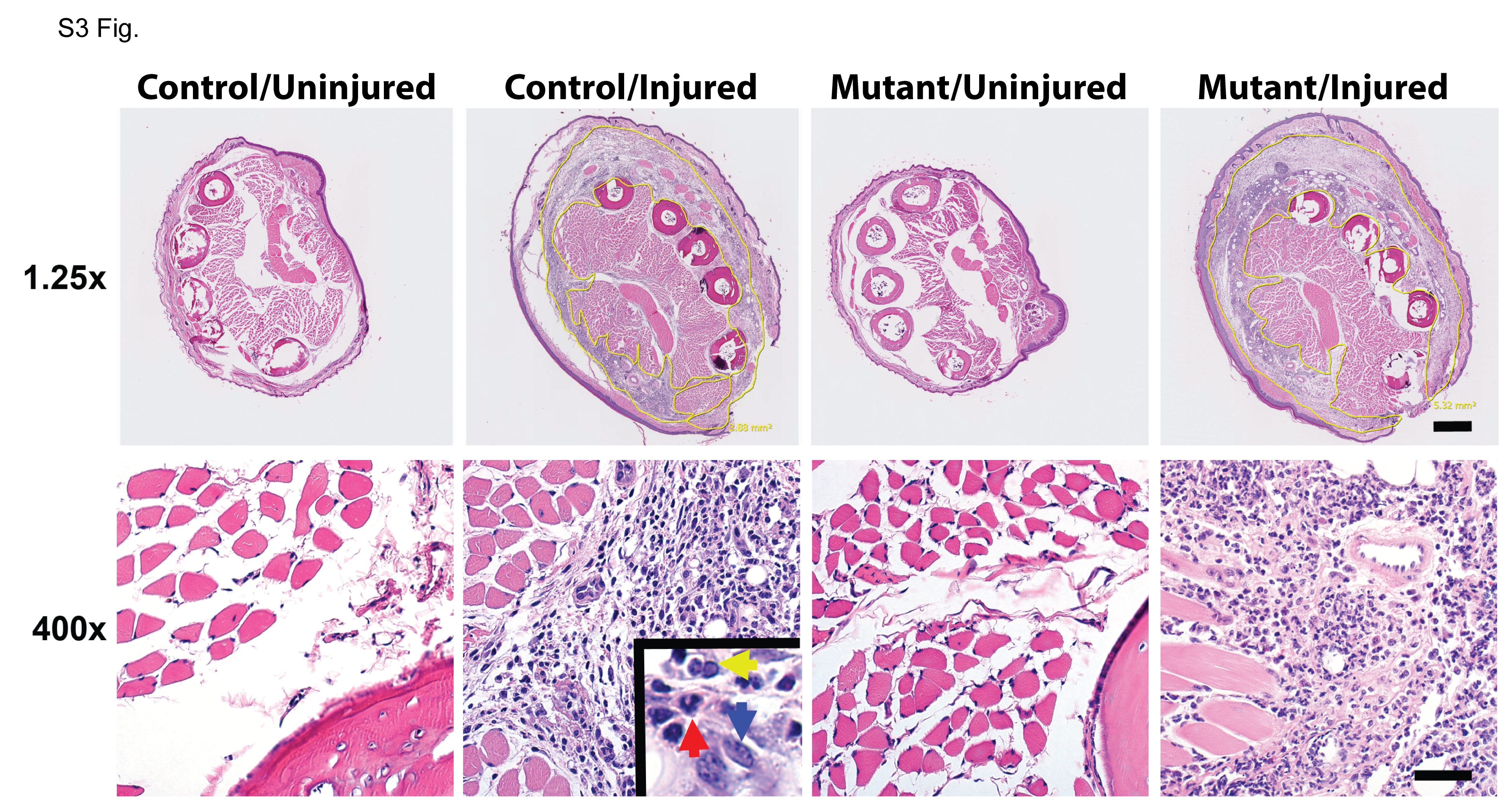

Supplement: S3 Fig — Cross-sections of PBS-injected (uninjured) or CFA-injected (injured) paws from both control and mutant mice were obtained. In injured tissues, the thin yellow border delineates the large area of inflammation within the CFA-injected paw. As shown, this was present in both the control and mutant paw. As evidenced in the 400x images, both control and mutant mice exhibited a mixed inflammatory infiltrate. The magnified inset identifies these components; lymphocytes (yellow arrow), neutrophils (red arrow), and macrophages (blue arrow) were present. (TIF) [file pone.0151602.s003.tif]

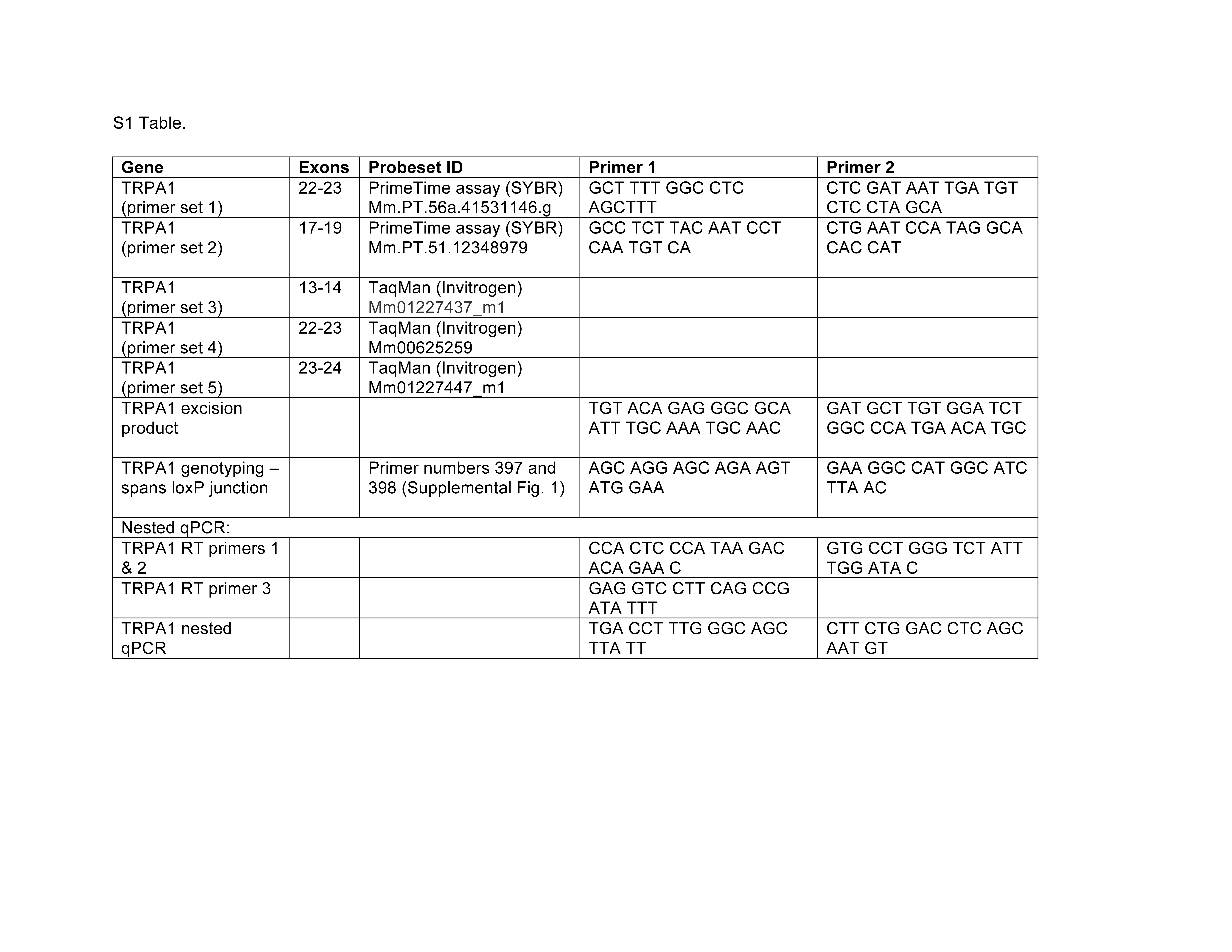

Supplement: S1 Table — (TIF) [file pone.0151602.s004.tif]

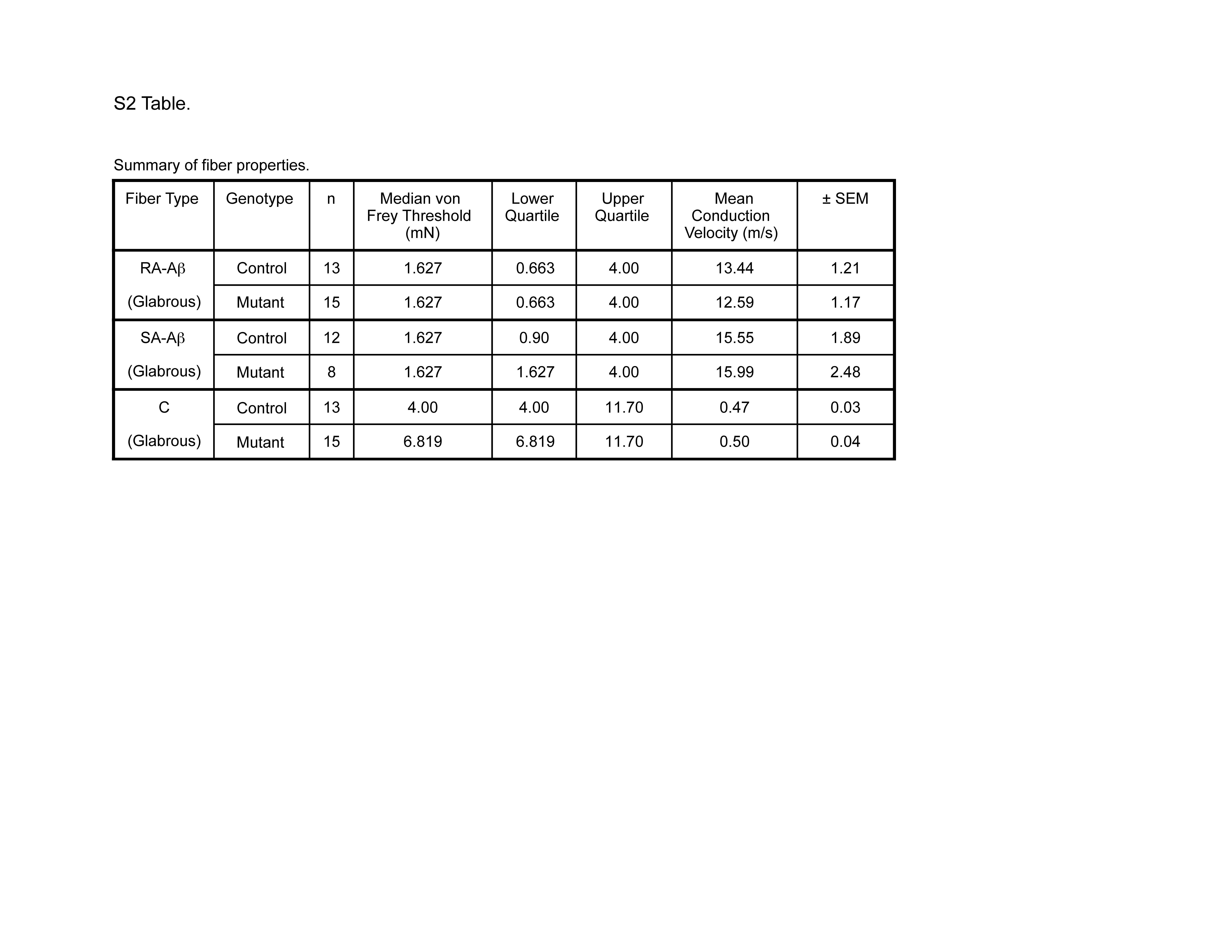

Supplement: S2 Table — (TIF) [file pone.0151602.s005.tif]
